# Changes in symptomatology, reinfection, and transmissibility associated with the SARS-CoV-2 variant B.1.1.7: an ecological study

**DOI:** 10.1016/S2468-2667(21)00055-4

**Published:** 2021-04-12

**Authors:** Mark S Graham, Carole H Sudre, Anna May, Michela Antonelli, Benjamin Murray, Thomas Varsavsky, Kerstin Kläser, Liane S Canas, Erika Molteni, Marc Modat, David A Drew, Long H Nguyen, Lorenzo Polidori, Somesh Selvachandran, Christina Hu, Joan Capdevila, COVID-19 Genomics UK (COG-UK) Consortium, Cherian Koshy, Amy Ash, Emma Wise, Nathan Moore, Matilde Mori, Nick Cortes, Jessica Lynch, Stephen Kidd, Derek J Fairley, Tanya Curran, James P McKenna, Helen Adams, Christophe Fraser, Tanya Golubchik, David Bonsall, Mohammed O Hassan-Ibrahim, Cassandra S Malone, Benjamin J Cogger, Michelle Wantoch, Nicola Reynolds, Ben Warne, Joshua Maksimovic, Karla Spellman, Kathryn McCluggage, Michaela John, Robert Beer, Safiah Afifi, Sian Morgan, Angela Marchbank, Anna Price, Christine Kitchen, Huw Gulliver, Ian Merrick, Joel Southgate, Martyn Guest, Robert Munn, Trudy Workman, Thomas R Connor, William Fuller, Catherine Bresner, Luke B Snell, Amita Patel, Themoula Charalampous, Gaia Nebbia, Rahul Batra, Jonathan Edgeworth, Samuel C Robson, Angela H Beckett, David M Aanensen, Anthony P Underwood, Corin A Yeats, Khalil Abudahab, Ben EW Taylor, Mirko Menegazzo, Gemma Clark, Wendy Smith, Manjinder Khakh, Vicki M Fleming, Michelle M Lister, Hannah C Howson-Wells, Louise Berry, Tim Boswell, Amelia Joseph, Iona Willingham, Carl Jones, Christopher Holmes, Paul Bird, Thomas Helmer, Karlie Fallon, Julian Tang, Veena Raviprakash, Sharon Campbell, Nicola Sheriff, Victoria Blakey, Lesley-Anne Williams, Matthew W Loose, Nadine Holmes, Christopher Moore, Matthew Carlile, Victoria Wright, Fei Sang, Johnny Debebe, Francesc Coll, Adrian W Signell, Gilberto Betancor, Harry D Wilson, Sahar Eldirdiri, Anita Kenyon, Thomas Davis, Oliver G Pybus, Louis du Plessis, Alex E Zarebski, Jayna Raghwani, Moritz UG Kraemer, Sarah Francois, Stephen W Attwood, Tetyana I Vasylyeva, Marina Escalera Zamudio, Bernardo Gutierrez, M. Estee Torok, William L Hamilton, Ian G Goodfellow, Grant Hall, Aminu S Jahun, Yasmin Chaudhry, Myra Hosmillo, Malte L Pinckert, Iliana Georgana, Samuel Moses, Hannah Lowe, Luke Bedford, Jonathan Moore, Susanne Stonehouse, Chloe L Fisher, Ali R Awan, John BoYes, Judith Breuer, Kathryn Ann Harris, Julianne Rose Brown, Divya Shah, Laura Atkinson, Jack CD Lee, Nathaniel Storey, Flavia Flaviani, Adela Alcolea-Medina, Rebecca Williams, Gabrielle Vernet, Michael R Chapman, Lisa J Levett, Judith Heaney, Wendy Chatterton, Monika Pusok, Li Xu-McCrae, Darren L Smith, Matthew Bashton, Gregory R Young, Alison Holmes, Paul Anthony Randell, Alison Cox, Pinglawathee Madona, Frances Bolt, James Price, Siddharth Mookerjee, Manon Ragonnet-Cronin, Fabricia F. Nascimento, David Jorgensen, Igor Siveroni, Rob Johnson, Olivia Boyd, Lily Geidelberg, Erik M Volz, Aileen Rowan, Graham P Taylor, Katherine L Smollett, Nicholas J Loman, Joshua Quick, Claire McMurray, Joanne Stockton, Sam Nicholls, Will Rowe, Radoslaw Poplawski, Alan McNally, Rocio T Martinez Nunez, Jenifer Mason, Trevor I Robinson, Elaine O'Toole, Joanne Watts, Cassie Breen, Angela Cowell, Graciela Sluga, Nicholas W Machin, Shazaad S Y Ahmad, Ryan P George, Fenella Halstead, Venkat Sivaprakasam, Wendy Hogsden, Chris J Illingworth, Chris Jackson, Emma C Thomson, James G Shepherd, Patawee Asamaphan, Marc O Niebel, Kathy K Li, Rajiv N Shah, Natasha G Jesudason, Lily Tong, Alice Broos, Daniel Mair, Jenna Nichols, Stephen N Carmichael, Kyriaki Nomikou, Elihu Aranday-Cortes, Natasha Johnson, Igor Starinskij, Ana da Silva Filipe, David L Robertson, Richard J Orton, Joseph Hughes, Sreenu Vattipally, Joshua B Singer, Seema Nickbakhsh, Antony D Hale, Louissa R Macfarlane-Smith, Katherine L Harper, Holli Carden, Yusri Taha, Brendan AI Payne, Shirelle Burton-Fanning, Sheila Waugh, Jennifer Collins, Gary Eltringham, Steven Rushton, Sarah O'Brien, Amanda Bradley, Alasdair Maclean, Guy Mollett, Rachel Blacow, Kate E Templeton, Martin P McHugh, Rebecca Dewar, Elizabeth Wastenge, Samir Dervisevic, Rachael Stanley, Emma J Meader, Lindsay Coupland, Louise Smith, Clive Graham, Edward Barton, Debra Padgett, Garren Scott, Emma Swindells, Jane Greenaway, Andrew Nelson, Clare M McCann, Wen C Yew, Monique Andersson, Timothy Peto, Anita Justice, David Eyre, Derrick Crook, Tim J Sloan, Nichola Duckworth, Sarah Walsh, Anoop J Chauhan, Sharon Glaysher, Kelly Bicknell, Sarah Wyllie, Scott Elliott, Allyson Lloyd, Robert Impey, Nick Levene, Lynn Monaghan, Declan T Bradley, Tim Wyatt, Elias Allara, Clare Pearson, Husam Osman, Andrew Bosworth, Esther Robinson, Peter Muir, Ian B Vipond, Richard Hopes, Hannah M Pymont, Stephanie Hutchings, Martin D Curran, Surendra Parmar, Angie Lackenby, Tamyo Mbisa, Steven Platt, Shahjahan Miah, David Bibby, Carmen Manso, Jonathan Hubb, Meera Chand, Gavin Dabrera, Mary Ramsay, Daniel Bradshaw, Alicia Thornton, Richard Myers, Ulf Schaefer, Natalie Groves, Eileen Gallagher, David Lee, David Williams, Nicholas Ellaby, Ian Harrison, Hassan Hartman, Nikos Manesis, Vineet Patel, Chloe Bishop, Vicki Chalker, Juan Ledesma, Katherine A Twohig, Matthew T.G. Holden, Sharif Shaaban, Alec Birchley, Alexander Adams, Alisha Davies, Amy Gaskin, Amy Plimmer, Bree Gatica-Wilcox, Caoimhe McKerr, Catherine Moore, Chris Williams, David Heyburn, Elen De Lacy, Ember Hilvers, Fatima Downing, Giri Shankar, Hannah Jones, Hibo Asad, Jason Coombes, Joanne Watkins, Johnathan M Evans, Laia Fina, Laura Gifford, Lauren Gilbert, Lee Graham, Malorie Perry, Mari Morgan, Matthew Bull, Michelle Cronin, Nicole Pacchiarini, Noel Craine, Rachel Jones, Robin Howe, Sally Corden, Sara Rey, Sara Kumziene-SummerhaYes, Sarah Taylor, Simon Cottrell, Sophie Jones, Sue Edwards, Justin O'Grady, Andrew J Page, Alison E Mather, David J Baker, Steven Rudder, Alp Aydin, Gemma L Kay, Alexander J Trotter, Nabil-Fareed Alikhan, Leonardo de Oliveira Martins, Thanh Le-Viet, Lizzie Meadows, Anna Casey, Liz Ratcliffe, David A Simpson, Zoltan Molnar, Thomas Thompson, Erwan Acheson, Jane AH Masoli, Bridget A Knight, Sian Ellard, Cressida Auckland, Christopher R Jones, Tabitha W Mahungu, Dianne Irish-Tavares, Tanzina Haque, Jennifer Hart, Eric Witele, Melisa Louise Fenton, Ashok Dadrah, Amanda Symmonds, Tranprit Saluja, Yann Bourgeois, Garry P Scarlett, Katie F Loveson, Salman Goudarzi, Christopher Fearn, Kate Cook, Hannah Dent, Hannah Paul, David G Partridge, Mohammad Raza, Cariad Evans, Kate Johnson, Steven Liggett, Paul Baker, Stephen Bonner, Sarah Essex, Ronan A Lyons, Kordo Saeed, Adhyana I.K Mahanama, Buddhini Samaraweera, Siona Silveira, Emanuela Pelosi, Eleri Wilson-Davies, Rachel J Williams, Mark Kristiansen, Sunando Roy, Charlotte A Williams, Marius Cotic, Nadua Bayzid, Adam P Westhorpe, John A Hartley, Riaz Jannoo, Helen L Lowe, Angeliki Karamani, Leah Ensell, Jacqui A Prieto, Sarah Jeremiah, Dimitris Grammatopoulos, Sarojini Pandey, Lisa Berry, Katie Jones, Alex Richter, Andrew Beggs, Angus Best, Benita Percival, Jeremy Mirza, Oliver Megram, Megan Mayhew, Liam Crawford, Fiona Ashcroft, Emma Moles-Garcia, Nicola Cumley, Colin P Smith, Giselda Bucca, Andrew R Hesketh, Beth Blane, Sophia T Girgis, Danielle Leek, Sushmita Sridhar, Sally Forrest, Claire Cormie, Harmeet K Gill, Joana Dias, Ellen E Higginson, Mailis Maes, Jamie Young, Leanne M Kermack, Ravi Kumar Gupta, Catherine Ludden, Sharon J Peacock, Sophie Palmer, Carol M Churcher, Nazreen F Hadjirin, Alessandro M Carabelli, Ellena Brooks, Kim S Smith, Katerina Galai, Georgina M McManus, Chris Ruis, Rose K Davidson, Andrew Rambaut, Thomas Williams, Carlos E Balcazar, Michael D Gallagher, Áine O'Toole, Stefan Rooke, Verity Hill, Kathleen A Williamson, Thomas D Stanton, Stephen L Michell, Claire M Bewshea, Ben Temperton, Michelle L Michelsen, Joanna Warwick-Dugdale, Robin Manley, Audrey Farbos, James W Harrison, Christine M Sambles, David J Studholme, Aaron R Jeffries, Alistair C Darby, Julian A Hiscox, Steve Paterson, Miren Iturriza-Gomara, Kathryn A Jackson, Anita O Lucaci, Edith E Vamos, Margaret Hughes, Lucille Rainbow, Richard Eccles, Charlotte Nelson, Mark Whitehead, Lance Turtle, Sam T Haldenby, Richard Gregory, Matthew Gemmell, Claudia Wierzbicki, Hermione J Webster, Thushan I de Silva, Nikki Smith, Adrienn Angyal, Benjamin B Lindsey, Danielle C Groves, Luke R Green, Dennis Wang, Timothy M Freeman, Matthew D Parker, Alexander J Keeley, Paul J Parsons, Rachel M Tucker, Rebecca Brown, Matthew Wyles, Max Whiteley, Peijun Zhang, Marta Gallis, Stavroula F Louka, Chrystala Constantinidou, Meera Unnikrishnan, Sascha Ott, Jeffrey K.J. Cheng, Hannah E. Bridgewater, Lucy R. Frost, Grace Taylor-Joyce, Richard Stark, Laura Baxter, Mohammad T. Alam, Paul E Brown, Dinesh Aggarwal, Alberto C Cerda, Tammy V Merrill, Rebekah E Wilson, Patrick C McClure, Joseph G Chappell, Theocharis Tsoleridis, Jonathan Ball, David Buck, John A Todd, Angie Green, Amy Trebes, George MacIntyre-Cockett, Mariateresa de Cesare, Alex Alderton, Roberto Amato, Cristina V Ariani, Mathew A Beale, Charlotte Beaver, Katherine L Bellis, Emma Betteridge, James Bonfield, John Danesh, Matthew J Dorman, Eleanor Drury, Ben W Farr, Luke Foulser, Sonia Goncalves, Scott Goodwin, Marina Gourtovaia, Ewan M Harrison, David K Jackson, Dorota Jamrozy, Ian Johnston, Leanne Kane, Sally Kay, Jon-Paul Keatley, Dominic Kwiatkowski, Cordelia F Langford, Mara Lawniczak, Laura Letchford, Rich Livett, Stephanie Lo, Inigo Martincorena, Samantha McGuigan, Rachel Nelson, Steve Palmer, Naomi R Park, Minal Patel, Liam Prestwood, Christoph Puethe, Michael A Quail, Shavanthi Rajatileka, Carol Scott, Lesley Shirley, John Sillitoe, Michael H Spencer Chapman, Scott AJ Thurston, Gerry Tonkin-Hill, Danni Weldon, Diana Rajan, Iraad F Bronner, Louise Aigrain, Nicholas M Redshaw, Stefanie V Lensing, Robert Davies, Andrew Whitwham, Jennifier Liddle, Kevin Lewis, Jaime M Tovar-Corona, Steven Leonard, Jillian Durham, Andrew R Bassett, Shane McCarthy, Robin J Moll, Keith James, Karen Oliver, Alex Makunin, Jeff Barrett, Rory N Gunson, Alexander Hammers, Andrew T Chan, Jonathan Wolf, Tim D Spector, Claire J Steves, Sebastien Ourselin

**Affiliations:** a School of Biomedical Engineering and Imaging Sciences, King's College London, London, UK; b Department of Twin Research and Genetic Epidemiology, King's College London, London, UK; c MRC Unit for Lifelong Health and Ageing, Department of Population Science and Experimental Medicine, University College London, London, UK; d Centre for Medical Image Computing, Department of Computer Science, University College London, London, UK; e Zoe Global, London, UK; f Clinical and Translational Epidemiology Unit, Massachusetts General Hospital and Harvard Medical School, Boston, MA, USA

## Abstract

**Background:**

The SARS-CoV-2 variant B.1.1.7 was first identified in December, 2020, in England. We aimed to investigate whether increases in the proportion of infections with this variant are associated with differences in symptoms or disease course, reinfection rates, or transmissibility.

**Methods:**

We did an ecological study to examine the association between the regional proportion of infections with the SARS-CoV-2 B.1.1.7 variant and reported symptoms, disease course, rates of reinfection, and transmissibility. Data on types and duration of symptoms were obtained from longitudinal reports from users of the COVID Symptom Study app who reported a positive test for COVID-19 between Sept 28 and Dec 27, 2020 (during which the prevalence of B.1.1.7 increased most notably in parts of the UK). From this dataset, we also estimated the frequency of possible reinfection, defined as the presence of two reported positive tests separated by more than 90 days with a period of reporting no symptoms for more than 7 days before the second positive test. The proportion of SARS-CoV-2 infections with the B.1.1.7 variant across the UK was estimated with use of genomic data from the COVID-19 Genomics UK Consortium and data from Public Health England on spike-gene target failure (a non-specific indicator of the B.1.1.7 variant) in community cases in England. We used linear regression to examine the association between reported symptoms and proportion of B.1.1.7. We assessed the Spearman correlation between the proportion of B.1.1.7 cases and number of reinfections over time, and between the number of positive tests and reinfections. We estimated incidence for B.1.1.7 and previous variants, and compared the effective reproduction number, R_t_, for the two incidence estimates.

**Findings:**

From Sept 28 to Dec 27, 2020, positive COVID-19 tests were reported by 36 920 COVID Symptom Study app users whose region was known and who reported as healthy on app sign-up. We found no changes in reported symptoms or disease duration associated with B.1.1.7. For the same period, possible reinfections were identified in 249 (0·7% [95% CI 0·6–0·8]) of 36 509 app users who reported a positive swab test before Oct 1, 2020, but there was no evidence that the frequency of reinfections was higher for the B.1.1.7 variant than for pre-existing variants. Reinfection occurrences were more positively correlated with the overall regional rise in cases (Spearman correlation 0·56–0·69 for South East, London, and East of England) than with the regional increase in the proportion of infections with the B.1.1.7 variant (Spearman correlation 0·38–0·56 in the same regions), suggesting B.1.1.7 does not substantially alter the risk of reinfection. We found a multiplicative increase in the R_t_ of B.1.1.7 by a factor of 1·35 (95% CI 1·02–1·69) relative to pre-existing variants. However, R_t_ fell below 1 during regional and national lockdowns, even in regions with high proportions of infections with the B.1.1.7 variant.

**Interpretation:**

The lack of change in symptoms identified in this study indicates that existing testing and surveillance infrastructure do not need to change specifically for the B.1.1.7 variant. In addition, given that there was no apparent increase in the reinfection rate, vaccines are likely to remain effective against the B.1.1.7 variant.

**Funding:**

Zoe Global, Department of Health (UK), Wellcome Trust, Engineering and Physical Sciences Research Council (UK), National Institute for Health Research (UK), Medical Research Council (UK), Alzheimer's Society.

## Introduction

In early December, 2020, a phylogenetically distinct cluster of SARS-CoV-2 was genetically characterised in the southeast of England. Most cases had been detected in November, with a small number detected as early as September, 2020.^1^ Genomic surveillance revealed that this new variant, termed B.1.1.7, has several mutations of immunological significance and has been spreading rapidly, with cases increasing in frequency.^2^ It is important to understand how these mutations could affect the presentation and spread of COVID-19 so that effective public health responses can be formulated.^3^

**Research in context**

**Evidence before this study**

To identify existing evidence on the SARS-CoV-2 B.1.1.7 variant, we searched PubMed and Google Scholar for articles published between Dec 1, 2020, and Feb 1, 2021, using the keywords “COVID-19” AND “B.1.1.7” with no language restrictions, finding 281 results. We did not find any studies that investigated B.1.1.7-associated changes in symptoms or their severity and duration, but found one study showing that the B.1.1.7 variant did not change the ratio of symptomatic to asymptomatic infections. We found six articles describing laboratory-based investigations of the responses of the B.1.1.7 variant to vaccine-induced immunity, but no work investigating what this means for natural immunity and the likelihood of reinfection outside the laboratory. We found five articles that showed increased transmissibility of the B.1.1.7 variant. Other identified studies were not relevant.

**Added value of this study**

To our knowledge, this is the first study to explore changes in symptom type and duration and community reinfection rates associated with the B.1.1.7 variant. We used self-reported symptom logs from 36 920 users of the COVID Symptom Study app who reported positive test results between Sept 28 and Dec 27, 2020. The B.1.1.7 variant was not associated with changes in the COVID-19 symptoms reported, nor their duration. We also did not find evidence for an increase in reinfections in the presence of the B.1.1.7 variant. We found a multiplicative increase in the effective reproduction number, R_t_, of the B.1.1.7 variant by a factor of 1·35 (95% CI 1·02–1·69) compared with pre-existing variants. However, we found that R_t_ fell below 1 during regional and national lockdowns, even in regions with high proportions of infections with the B.1.1.7 variant.

**Implications of all the available evidence**

Our findings suggest that existing criteria for symptomatic COVID-19 testing need not change as a result of the increase in infections with the B.1.1.7 variant. Building on the results of laboratory studies, the finding that reinfection is not more likely in the presence of the B.1.1.7 variant suggests that immunity developed from infection with pre-existing variants is likely to protect against the B.1.1.7 variant and that vaccines will probably remain effective against this new variant. Our results add to the emerging consensus that the B.1.1.7 variant has increased transmissibility. The finding that R_t_ fell below 1 during regional and national lockdowns, even in regions with high levels of infection with the B.1.1.7 variant, requires further investigation to establish the factors that enabled this decrease and thus to inform countries seeking to control the spread of the B.1.1.7 variant.

Preliminary evidence from epidemiological studies suggests that the B.1.1.7 variant is more transmissible than pre-existing variants. Davies and colleagues^4^ found the B.1.1.7 variant to be 43–90% (95% CI 38–130) more transmissible than pre-existing variants, and Volz and colleagues have shown that the B.1.1.7 variant increases the effective reproduction number, R_t_, by a factor of 1·5–2·0.^5^ Evidence suggests that the B.1.1.7 variant increases the risk of admission to hospital and death.^6^ However, much is still unknown. From a public health perspective, it is crucial to understand whether the B.1.1.7 variant necessitates changes to existing measures for disease monitoring and containment. For instance, changes to symptomatology could require modifications to symptomatic testing programmes to ensure that new cases are identified, and changes to disease duration could require changes in the duration of isolation required for infected individuals. It is important for modelling and forecasting to understand whether the B.1.1.7 variant alters the rate of reinfection. Early estimates of the transmissibility of the B.1.1.7 variant are uncertain and additional estimates using independent data sources are needed. Furthermore, it is important to understand how these findings will affect measures to control the spread of the pandemic using non-pharmaceutical interventions, such as lockdowns.

We aimed to investigate the symptomatology, disease course, rates of reinfection, and transmissibility of the B.1.1.7 variant in the UK population.

## Methods

### Study design and participants

We did ecological studies to assess the symptoms, disease course, rates of reinfection, and transmissibility associated with increasing proportions of infections with the B.1.1.7 variant in the UK population. We used data from the COVID Symptom Study,^7^ a longitudinal dataset providing symptom reports and test results from a population of more than 4 million adults living in the UK, in combination with surveillance data from the COVID-19 Genomics UK (COG-UK) Consortium^8^ and a spike-gene target failure (SGTF) correlate in community testing data.

The study was approved by the King's College London Ethics Committee (REMAS ID 18210, review reference LRS-19/20-18210). All participants provided consent through the COVID Symptom Study app.

### Data sources

Longitudinal data were prospectively collected through the COVID Symptom Study app, developed by Zoe Global with input from King's College London (London, UK), Massachusetts General Hospital (Boston, MA, USA), and Lund and Uppsala Universities (Sweden). The app^7^ guides users through a set of enrolment questions, establishing baseline demographic and health information. Users are asked to record each day whether they feel physically normal and, if not, to log any symptoms. After a user reports any symptoms, they are asked “Where are you right now?”, with the options “At home”, “At hospital with suspected COVID-19 symptoms”, or “Back from hospital”. Users are also asked to maintain a record of any COVID-19 tests and their date, type, and result in the app. Users are able to record the same data on behalf of others, such as family members, to increase data coverage among those unlikely to use mobile apps, such as older adults. We included users living in the UK who had logged responses through the app at least once in the period between Sept 28 and Dec 27, 2020.

We used data released on Jan 13, 2021, from COG-UK to extract time-series of the percentage of daily cases resulting from the B.1.1.7 variant in Scotland, Wales, and each of the seven National Health Service (NHS) regions in England. Northern Ireland was excluded because of the low number of samples in the COG-UK dataset. These data were produced by sequencing a sample of PCR tests done in the community. Because of a delay of around 2 weeks^2^ between PCR tests and genomic sequencing, we used data only from samples taken up to Dec 31, 2020, to avoid censoring effects.

Additionally, we used data from Public Health England on the probable new variant captured in community cases in England according to SGTF. One of the spike gene mutations in the B.1.1.7 variant has been observed to cause an SGTF in the test used in three of England's large laboratories for the analysis of community cases.^1^ This failure results in a marker that is sensitive, but not necessarily specific, to the B.1.1.7 variant, as other circulating variants also contain the mutation leading to an SGTF. Comparison with genomic data shows that, from Nov 30, 2020, onwards, more than 96% of cases with the SGTF were from the B.1.1.7 variant.^9^ The proportion of SGTF cases is made available in England for each of the 316 lower-tier local authorities. We grouped these data into each NHS region using a population-weighted average to enable integration with other data sources.

### Statistical analysis

To assess whether the symptomatology of infection with the B.1.1.7 variant differed from that of previous variants, we investigated the change in symptom reporting from Sept 28 to Dec 27, 2020, covering 13 complete weeks over the period when the proportion of infections with the B.1.1.7 variant grew most notably in the NHS regions of London, South East, and East of England. For each week, in every region considered, we calculated the proportion of users reporting each symptom. Users were included in a week if they had reported a positive swab result (by PCR or lateral-flow test) in the period 14 days before or after that week. For each region and symptom, we did a linear regression, examining the association between infections and the B.1.1.7 variant as a proportion of total SARS-CoV-2 infections in that region (independent variable) and the proportion of users reporting the symptom (dependent variable) over the 13 weeks considered. We adjusted for the age and sex of users, as well as for two seasonal environmental confounders: regional temperature and humidity. Seasonal confounders were calculated each day from the temperature and relative humidity at 2 m above the surface (obtained from NASA climate data), averaged across each region considered.

We also examined the association between the proportion of infections with the B.1.1.7 variant and the disease burden, measured here as the total number of different symptoms reported over a period of 2 weeks before and 2 weeks after the test, and the relation with asymptomatic infection, defined as users reporting a positive test result but no symptoms in the 2 weeks before or after the test. We also investigated the rate of self-reported hospital visits, including users who reported being in hospital with suspected COVID-19 symptoms or being back from hospital. We also investigated the proportion of individuals reporting a long duration of symptoms, using a previously published definition of continuous symptoms reported for at least 28 days.^10^ To avoid censoring effects, the analyses of admission to hospital and long symptom duration included symptom reports to Jan 18, 2021, and the analysis of long symptom duration also considered reports of positive tests up to Dec 21, 2020. All analyses were adjusted for sex, age, temperature, and humidity. We controlled for the false discovery rate to account for multiple comparisons.

We defined possible reinfection as the presence of two reported positive tests separated by more than 90 days with a period of reporting no symptoms for more than 7 days before the second positive test. We calculated the proportion of possible reinfections among individuals who reported their first positive test before Oct 1, 2020. To assess whether the risk of reinfection was stronger in the presence of the B.1.1.7 variant, we did ecological studies in every region, examining the Spearman correlation between the proportion of infections with the B.1.1.7 variant and the number of reinfections over time, and between the proportion of positive tests reported through the app and the number of reinfections. We compared these two correlations in each region with use of the Mann-Whitney *U* test.

Daily estimates of the incidence of SARS-CoV-2 infection in Scotland, Wales, and each of the seven NHS regions in England during the period from Oct 1 to Dec 27, 2020, were produced using data from the COVID Symptom Study app and previously described methods.^11^ Using the COG-UK data to estimate the proportion of infections with the B.1.1.7 variant in each region per day, these incidence estimates were decomposed into two incidence time-series per region, one for pre-existing variants and one for B.1.1.7, with the constraint that the two time-series should sum to match the total incidence. R_t_ was estimated separately for the pre-existing variants and B.1.1.7 using previously described methods.^11^ Briefly, we used the relationship I_t+1_=I_t_exp(μ[R_t_ – 1]), where 1/μ is the serial interval and I_t_ the incidence on day t. We modelled the system as a Poisson process and assumed that the serial interval was drawn from a gamma distribution with α=6·0 and β=1·5, and used Markov Chain Monte-Carlo methods to estimate R_t_. We compared both multiplicative and additive differences of the new and old R_t_ values for days when the proportion of infections with the B.1.1.7 variant in a region was greater than 3%. Although data were not available for the proportion of infections with the B.1.1.7 variant in January, 2021, we also computed total incidence and R_t_ from Oct 1, 2020, to Jan 16, 2021, to see how they changed during the national lockdown in England.

### Role of the funding source

Zoe Global developed the app for data collection. The funders of the study had no role in the study design, data collection, data analysis, data interpretation, or writing of the report.

## Results

From March 24 to Dec 27, 2020, 4 327 245 participants from the UK signed up to use the COVID Symptom Study app. We excluded users living in Northern Ireland because of the low number of users who signed up (38 976 users), as well as 383 352 users without information on sex, and 2 175 979 who had not logged responses in the app between Sept 28 and Dec 27, 2020, leaving a total of 1 767 914 users. From Sept 28 to Dec 27, these users collectively recorded 65 613 697 logs in the app. In the same period, 497 989 users reported a swab test, of whom 55 192 reported a positive test, and we investigated the symptom reports of the 36 920 of those with a positive test whose region was known and who reported as healthy on app sign-up. The table shows the demographic data for the cohort studied.

**Table:** Characteristics of COVID Symptom Study app users active between Sept 28 and Dec 27, 2020

	**Overall**	**Tested**	Tested positive*	**Signed up healthy, with reporting around positive test**
**Total**				
Users	1 767 914	497 989	55 192	40 463
Daily reports†	65 613 697	19 154 601	1 514 244	1 497 061
**Age, years**				
Mean (SD)	48·4 (19·3)	46·06 (17·8)	42·1 (16·8)	42·9 (17·0)
≤18	163 112 (9·2%)	40 717 (8·2%)	5468 (9·9%)	3874 (9·6%)
19–64	1 234 259 (69·8%)	381 900 (76·7%)	45 149 (81·8%)	32 878 (81·3%)
≥65	370 543 (21·0%)	72 741 (14·6%)	4367 (7·9%)	3600 (8·9%)
Invalid	5576 (0·3%)	2631 (0·5%)	208 (0·4%)	111 (0·3%)
**Sex**				
Female	1 046 074 (59·2%)	315 875 (63·4%)	34 516 (62·5%)	24 844 (61·4%)
Male	720 562 (40·8%)	181 110 (36·4%)	20 546 (37·2%)	15 545 (38·4%)
Intersex	79 (<0·1%)	21 (<0·1%)	3 (<0·1%)	3 (<0·1%)
Prefer not to say	1199 (0·1%)	983 (0·2%)	127 (0·2%)	71 (0·2%)
**Region**				
South East	342 881 (19·4%)	97 143 (19·5%)	8762 (15·9%)	6555 (16·2%)
East of England	196 063 (11·1%)	57 680 (11·6%)	5373 (9·7%)	4037 (10%)
London	227 004 (12·8%)	81 940 (16·5%)	9733 (17·6%)	7384 (18·2%)
Midlands	198 350 (11·2%)	57 582 (11·6%)	6695 (12·1%)	4756 (11·8%)
North East and Yorkshire	156 999 (8·9%)	42 986 (8·6%)	5292 (9·6%)	3744 (9·3%)
North West	123 201 (7%)	45 156 (9·1%)	6180 (11·2%)	4399 (10·9%)
South West	186 372 (10·5%)	46 780 (9·4%)	3685 (6·7%)	2637 (6·5%)
Scotland	872 63 (4·9%)	13 793 (2·8%)	1589 (2·9%)	1049 (2·6%)
Wales	828 86 (4·7%)	16 471 (3·3%)	3092 (5·6%)	2359 (5·8%)
Not known	165 164 (9·3%)	38 458 (7·7%)	4638 (8·4%)	3543 (8·8%)

Data are n or n (%) unless otherwise specified. Invalid age refers to ages <1 or >100, which were usually caused by incorrect entries (eg, confusing the date of birth field with age).

* There could be more than one test per individual as the overall number contains failed tests and unknown results.

† Reports logged between Sept 28 and Dec 27, 2020; for some analyses we took further reports from an extended period from Sept 14, 2020, to Jan 18, 2021.

**Figure 1:**
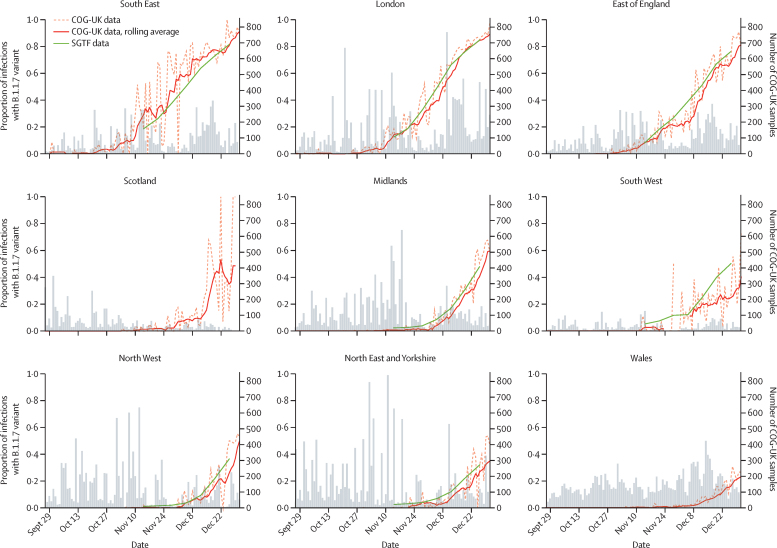
Presence of the B.1.1.7 variant by region in the UK from Sept 27 to Dec 31, 2020, measured using COG-UK genomic surveillance data and SGTF data Grey bars indicate the daily number of samples of positive cases available in the COG-UK data. SGTF data were not available for Scotland or Wales. COG-UK=COVID-19 Genomics UK Consortium. SGTF=spike-gene target failure.

Between Sept 27 and Dec 31, 2020, 98 170 sequences were made available by COG-UK, corresponding to 4·4% of the 2 207 476 cases recorded during this period.^12^ 16 224 (16·5%) sequences were of variant B.1.1.7. Considering the mean of the rolling average across December, 2020, the three regions with the largest proportion of infections with the B.1.1.7 variant were the South East, London, and East of England. The three regions with the lowest proportion were Wales, the North East and Yorkshire, and the North West. SGTF data were made available in England on a weekly basis from Nov 10 to Dec 29, 2020. Of the 700 590 cases reported during this period, 295 404 (42·2%) caused an SGTF. Examining the COG-UK data from England in the same period, we found 14 074 (34·6%) of 40 648 cases to be caused by the B.1.1.7 variant. The difference is in part attributable to the SGTF being a non-specific marker of B.1.1.7: according to Public Health England,^2^ in the week of Nov 9–15, 79% of cases with an SGTF were due to B.1.1.7, and from Nov 30 at least 96% of cases with an SGTF were caused by the B.1.1.7 variant. Figure 1 shows the increase in the proportion of infections with the B.1.1.7 variant over time in regions of the UK, using the COG-UK and SGTF data.

**Figure 2:**
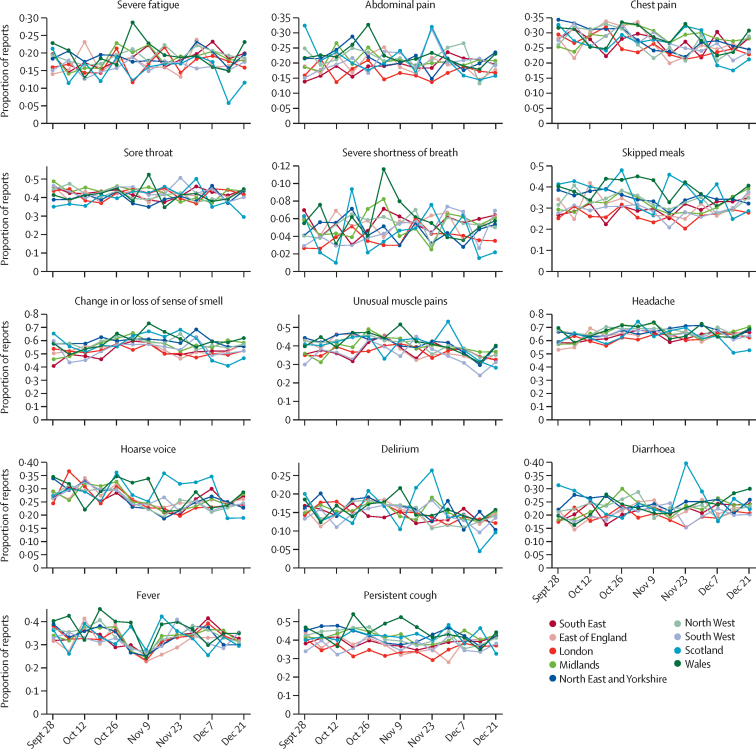
Regional plots of the frequency of reporting of each symptom in users reporting positive test results over time from Sept 28 to Dec 27, 2020 Symptom occurrence per 1-week window is shown smoothed over three timepoints as a function of time. The drop in reporting of fever in early November was caused by a change in the wording of the question; this wording was subsequently reverted a week later.

Analysis of the variation in symptom occurrence over time showed no qualitative change in the proportion of users reporting each symptom with an increasing proportion of infections with the B.1.1.7 variant (figure 2; appendix p 2). Linear regression (both unadjusted and adjusted for participant age and sex, and regional temperature and humidity) did not show evidence of an association between the proportion of infections with the B.1.1.7 variant and symptoms reported, after controlling for the false discovery rate (appendix p 6).

Visual inspection of the total number of symptoms reported, asymptomatic infections, self-reported hospital visits, and instances of long symptom duration over time suggested no change in any of these outcomes with an increasing proportion of infections with the B.1.1.7 variant (appendix pp 3–4). When correcting for mean age, sex, and ambient temperature and humidity, there was no evidence of an association between the proportion of infections with the B.1.1.7 variant and the number of symptoms reported over a 4-week period, the number of admissions to hospital, long symptom duration, or the proportion of asymptomatic cases (appendix p 8).

**Figure 3:**
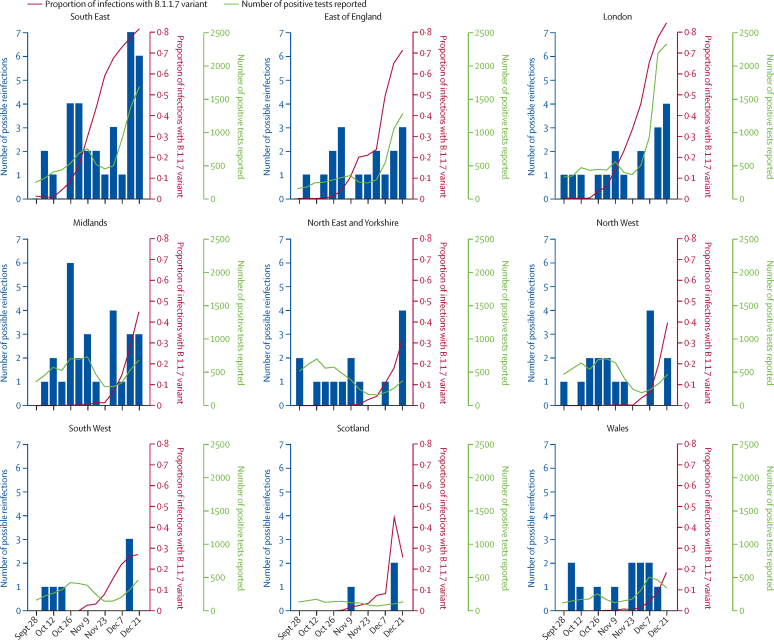
Number of reports of possible reinfection by region from Sept 28 to Dec 27, 2020 Bars indicate the number of reports of possible reinfection each week, plotted by the date of the second infection. Lines show the total number of positive tests reported through the COVID Symptom Study app and the proportion of infections with the B.1.1.7 variant for the same period.

We identified 304 individuals who reported two positive tests separated by an interval of at least 90 days. Among these individuals, symptom reporting allowed us to identify 249 users for whom there was a period of at least 7 symptom-free days in between positive tests, accounting for 0·7% (95% CI 0·6–0·8) of the 36 509 individuals who reported a positive swab test before Oct 1, 2020. Daily reports were available in the periods around both positive tests for 173 of those 249 users. There was no difference in reinfection reporting rates across the different NHS regions (p=0·11; appendix p 9). Figure 3 shows the evolution in the number of possible reinfections along with reported positive cases and the proportion of infections with the B.1.1.7 variant. For all regions except Scotland (which had a low number of app users), reinfection occurrences were more positively correlated with the overall regional rise in cases (r_s_=0·56 to 0·69 for South East, London, and East of England) than the regional rise in the proportion of infections with the B.1.1.7 variant (r_s_=0·38 to 0·56 for South East, London, and East of England; appendix p 9). Comparison of bootstrapped median values of these correlations using the Mann-Whitney *U* test showed the differences in correlation within each region were significant (p<0·001; appendix p 10).

**Figure 4:**
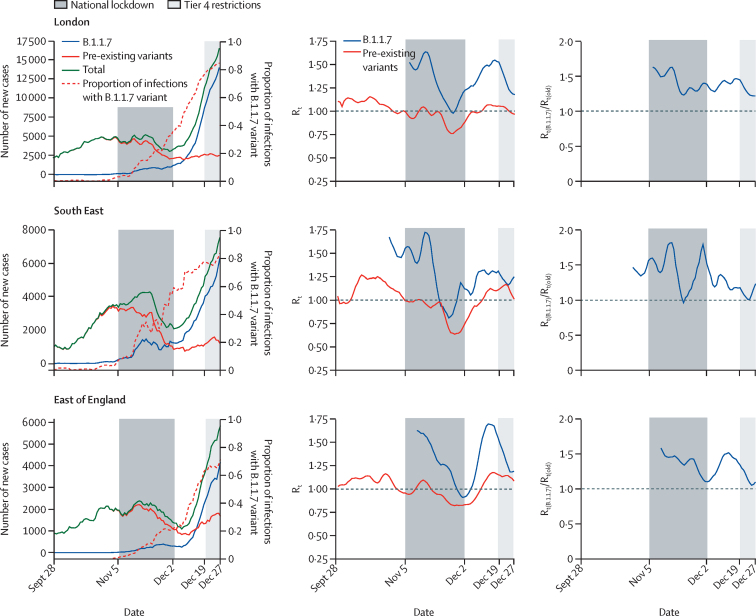
Total case numbers and R_t_ for pre-existing variants and B.1.1.7, and ratio between these R_t_ values, from Sept 28 to Dec 27, 2020 Data are shown for the three regions in England with the highest proportion of infections with the B.1.1.7 variant. R_t_=effective reproduction number.

When assessing the incidence and R_t_ for pre-existing variants and the B.1.1.7 variant in the three regions in England with the highest proportion of infections with the B.1.1.7 variant, the R_t_ of the B.1.1.7 variant was consistently greater than that of other variants (figure 4). The mean additive increase in R_t_ for the B.1.1.7 variant was 0·34 (95% CI 0·02–0·66), and the mean multiplicative increase was 1·35 (1·02–1·69). England exited its second national lockdown on Dec 2, 2020, leading to a change in behaviour and R_t_. When considering only the period after the end of the second lockdown, we found a mean additive increase in R_t_ of 0·28 (0·01–0·61) and a mean multiplicative increase of 1·28 (1·02–1·61) for the B.1.1.7 variant. Conducting the same analysis using SGTF data, limited to the period after Dec 1, 2020, when at least 95% of all SGTF cases were attributable to B.1.1.7, we found the R_t_ of the B.1.1.7 variant to have a mean additive increase of 0·26 (0·15–0·37) and a mean multiplicative increase of 1·25 (1·17–1·34; appendix p 5). These data were provided weekly, and linear interpolation was used to obtain daily estimates, leading to smoother estimates for variant-specific incidence and R_t_.

**Figure 5:**
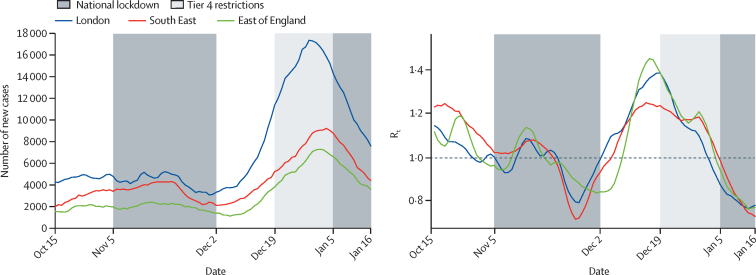
Total case numbers and R_t_ from Oct 15, 2020, to Jan 16, 2021, capturing the third national lockdown beginning Jan 5, 2021 Data are shown for the three regions in England with the highest proportion of infections with the B.1.1.7 variant in December, 2020. R_t_=effective reproduction number.

On Dec 19, 2020, London and much of the South East and East of England were placed under Tier 4 restrictions, enforcing stricter rules for physical distancing and decreased human-to-human contact that stopped short of nationwide measures. On Jan 5, 2021, the whole of England was placed in national lockdown. In January, the proportion of infections with the B.1.1.7 variant in London, the South East, and the East of England (these three regions had the largest proportion of infections with the B.1.1.7 variant in England) was at least 80%, assuming the proportion had not decreased from the end of December. R_t_ fell to around 0·8 in all three of these regions during the national lockdown (figure 5). An extended plot including Scotland, Wales, and all regions in England, is shown in the appendix (p 7).

## Discussion

Using data collected through community reporting of symptoms and tests via the COVID Symptom Study app, we did an ecological study to investigate whether the appearance of the B.1.1.7 variant, first detected in a sample from England in September, 2020, was associated with differences in symptoms, disease duration, admission to hospital, asymptomatic infection, risk of reinfection, and transmissibility for users reporting a positive test result between Sept 28 and Dec 27, 2020. We did not find associations between the proportion of infections with the B.1.1.7 variant and the type of symptoms reported by our app users. We also did not find evidence for any change associated with the B.1.1.7 variant in the total number of symptoms reported by individuals, nor in the proportion of individuals with a long disease duration, defined as recording symptoms for more than 28 days without a break of more than 7 days. The proportion of users with asymptomatic disease did not significantly change as the B.1.1.7 variant increased in prevalence, in agreement with other studies on the subject.^13^ We also found no changes in admissions to hospital; however, other reports have shown that the B.1.1.7 variant increases rates of admission to hospital.^6^

Limitations to the assessments of the proportions of asymptomatic cases and admission to hospital should be noted: most of our users get tested only when they have symptoms, so relatively few asymptomatic infections are recorded, and the self-reported nature of our data on admission to hospital means we are likely to miss more severe hospitalised cases, when the individual is unlikely to self-report. There is also evidence that infection with the B.1.1.7 variant is associated with increased risk of mortality,^6^ and our data do not allow us to assess this.

A report from the COVID-19 Infection Survey, conducted by the UK Office for National Statistics, showed that individuals infected with the B.1.1.7 variant were more likely to report a cough, sore throat, fatigue, myalgia, and fever in the 7 days preceding the test, and less likely to report a loss of taste or smell.^14^ It is not clear whether this report adjusted for age, sex, and environmental factors, although we found that adjustment for these factors did not affect the results of our analysis (appendix p 6). The discrepancy between our results and those of the COVID-19 Infection Survey might be explained by sampling at different points in the disease course. Our participants predominantly sought testing at symptom onset, whereas the design of the COVID-19 Infection Survey means that the tests could have been administered to the participants at any point during the disease course. The B.1.1.7 variant has been shown to cause longer infections,^15^ which means that symptom reports from COVID-19 Infection Survey participants infected with the B.1.1.7 variant might have been recorded later in the disease course than those infected with other variants, causing apparent differences. The periods considered also differed: we considered symptoms reported 2 weeks before and 2 weeks after the positive test result, in contrast to the 1 week before the positive test considered by the COVID-19 Infection Survey. Further opportunities to study symptoms associated with infection with the B.1.1.7 variant in different contexts are required to provide definitive answers.

We observed, among 249 potential cases, a very low prevalence of possible reinfection (0·7% [95% CI 0·6–0·8]), consistent with another study of 6614 health-care workers who had previously tested positive for COVID-19, in which 44 (0·66%) possible reinfections were identified.^16^ Our reinfection rate did not vary across regions or time, which is consistent with the hypothesis that reinfection is no more likely in the context of the B.1.1.7 variant. This might mean that, if adequate immunity is built during the first infection, it might be sufficient to protect against reinfection in the presence of the B.1.1.7 variant. Ultimately, this is a positive sign that the immunity built through vaccination against pre-existing variants could also be effective against the B.1.1.7 variant. This finding is in line with initial, laboratory-based studies of the efficacy of vaccines designed for pre-existing variants against this newer variant.^17^, ^18^, ^19^

We found an increase in R_t_ associated with the B.1.1.7 variant. There was a mean multiplicative increase in R_t_ of 1·35 (95% 1·02–1·69), which is similar to estimates from Volz and colleagues,^5^ who estimated an increase in R_t_ of 1·5–2·0; Davies and colleagues,^4^ who estimated an increase in transmissibility of 1·43–1·90 (95% CI 1·38–2·30); and Walker and colleagues,^13^ who found an increase in growth rate that corresponded to a transmissibility increase of 1·33 (95% CI 1·21–1·53) assuming a generation time of 4·7 days.^20^ These increases in transmissibility have worrying implications for the ability of lockdown measures to control spread of the B.1.1.7 variant, given that R_t_ was estimated to be 0·7–0·9 during the first national lockdown in England.^21^ However, we found R_t_ to be around 0·8 in the three regions in England in which at least 80% of infections were likely to be due to the B.1.1.7 variant during the national lockdown beginning on Jan 5, 2021. There are several potential explanations for this finding. Adherence to this lockdown could have been greater than in previous lockdowns, helping to reduce R_t_. The true increase in transmissibility might also be at the lower end of the available estimates, or it is possible that the increase in transmissibility estimated outside lockdown cannot be extrapolated to lockdown, perhaps because of the B.1.1.7 variant responding differently to lockdown measures than pre-existing variants. Another possible explanation is that there is now sufficient community immunity to reduce R_t_ further than in previous lockdowns. One serology study estimated that, from Dec 21, 2020, to Jan 18, 2021, 15·3% (95% CI 14·7–15·9) of individuals in England would have tested positive for COVID-19 antibodies.^22^ Many countries have now detected infections with the B.1.1.7 variant, and work to better understand the factors that helped to suppress its spread in the UK will help other countries to formulate their public health responses.^23^

Our study has several strengths. The large, longitudinal nature of the COVID Symptom Study data, with good coverage of the UK population, provides a unique opportunity to study potential changes in symptomatology and disease duration. The ability to match tests and symptom reports over long periods also allows us to measure possible reinfection rates. Our data also offer the ability to provide a valuable complementary measure to existing measurements of the increased transmissibility of the B.1.1.7 variant: we were able to use real-time, representative incidence estimates to measure R_t_, whereas other studies have relied on deaths and admissions to hospital, which are lagged, or community case numbers, which do not reflect true infection numbers.

We acknowledge several limitations to this study. First, as we had no information on the variant causing individual positive infections reported through the app, we did an ecological study, assessing the association between the proportion of infections with the B.1.1.7 variant and population-level measures. This design does not allow for causal interpretation of the effect of the B.1.1.7 variant on the measures we investigate. Our work assumes that all non-B.1.1.7 variants in circulation at the time of study give rise to the same range of symptoms and immune response, and have the same transmissibility. Genomic surveillance has detected a very low number of non-B.1.1.7 variants of concern in circulation,^24^ supporting the validity of this assumption, but it cannot be ruled out that other variants with different characteristics are circulating undetected.

Second, data obtained from participatory digital platforms have well documented^25^ biases in demographics. Although we were able to correct for some of these factors, such as age and sex, in our analysis, others are more difficult to characterise and correct for. For example, respondents signing up to a participatory platform such as the COVID Symptom Study app are likely to be more interested in health and COVID-19 than the wider population, and might exhibit different behaviours. Participatory studies might also suffer from ascertainment or collider bias.^26^ Self-report also carries the risk of data input errors, although the design of the app seeks to minimise this; for example, each time a user submits a log in the app, they are shown the full history of their test results and are given the option to amend incorrect entries. Previous publications from our group have found that population-level estimates of disease prevalence from our app triangulate well with those obtained from studies designed to be representative of the population.^11^

Another limitation was that we assumed that testing positive for SARS-CoV-2 infection after an interval of 90 days with at least a 7-day period with an absence of symptoms is consistent with reinfection. Repeated positive testing has been reported shortly after hospital discharge,^27^ with PCR positivity detected up to 28 days after symptom resolution. Although the chosen cutoff of 90 days between two positive tests is unlikely to be due to prolonged PCR positivity, this cannot be ruled out; however, this would probably only affect a small number of cases. Viral sequencing of the two infections would ideally be used to confirm reinfection.

Finally, despite correcting for changes in temperature and humidity, comparisons in symptoms were made over time, and seasonal effects (including effects on symptoms) might not have been fully taken into account.^28^

In summary, after examining the effect of the proportion of infections with the SARS-CoV-2 B.1.1.7 variant on COVID-19 symptoms, disease course, rates of reinfection, and transmissibility in the UK, we found no change in symptoms or their duration. Reinfections were rare (0·7% of app users) and there was no evidence of increased reinfection rates associated with the prevalence of the B.1.1.7 variant. We found an increase in R_t_ for the B.1.1.7 variant, but R_t_ fell below 1 during lockdown, even in regions with very high (>80%) proportions of infections with the B.1.1.7 variant.

## Data sharing

Data collected in the COVID Symptom Study smartphone app are being shared with other health researchers through the UK NHS-funded Health Data Research UK and Secure Anonymised Information Linkage consortium, housed in the UK Secure Research Platform (Swansea, UK). Anonymised data are available to be shared with researchers according to their protocols in the public interest (https://web.www.healthdatagateway.org/dataset/fddcb382-3051-4394-8436-b92295f14259). US investigators are encouraged to coordinate data requests through the Coronavirus Pandemic Epidemiology Consortium (https://www.monganinstitute.org/cope-consortium).

## Declaration of interests

AM, LP, SS, JC, CH, and JW are employees of Zoe Global. TDS is a consultant for Zoe Global. DAD and ATC previously served as investigators on a clinical trial of diet and lifestyle using a separate smartphone app supported by Zoe Global. ATC reports grants from the Massachusetts Consortium on Pathogen Readiness during the conduct of the study; and personal fees from Pfizer, Boehringer Ingelheim, and Bayer Pharma outside the submitted work. DAD reports grants from the US National Institutes of Health, the Massachusetts Consortium on Pathogen Readiness, and the American Gastroenterological Association during the conduct of the study. All other authors declare no competing interests.
