# Supplementary material for: Changes in symptomatology, reinfection, and transmissibility associated with the SARS-CoV-2 variant B.1.1.7: an ecological study
Source: Lancet Public Health. 2021 Apr 12;6(5):e335–45. doi: 10.1016/S2468-2667(21)00055-4 (PMC8041365; doi:10.1016/S2468-2667(21)00055-4)
Supplement: Supplementary appendix [file mmc1.pdf]

THE LANCET
Public Health

Supplementary appendix

This appendix formed part of the original submission and has been peer reviewed. We post it as supplied by the authors.

Supplement to: Graham MS, Sudre CH, May A, et al. Changes in symptomatology, reinfection, and transmissibility associated with the SARS-CoV-2 variant B.1.1.7: an ecological study. *Lancet Public Health* 2021; published online April 12. http://dx.doi.org/10.1016/S2468-2667(21)00055-4.

Changes in symptomatology, re-infection and transmissibility associated with SARS-CoV-2 variant B.1.1.7: an ecological study

Supplementary appendix

Supplementary Figures 2

Figure S1. Symptom reporting against proportion of B.1.1.7. 2

Figure S2. Hospitalisation, asymptomatics, long duration and total symptoms against time. 3

Figure S3. Hospitalisation, asymptomatics, long duration and total symptoms against proportion of B.1.1.7. 4

Figure S4. Incidence and R(t) using SGTF data. 5

Figure S5. Associations between symptom reports and B.1.1.7. 6

Figure S6. Incidence and R(t) for all regions, extended to 15 January 2021. 7

Supplementary Tables 8

Table S1. Coefficients for association between B.1.1.7 and asymptomatic infection, long duration, hospital reports, number of symptoms. 8

Table S2. Regional reinfection rates and correlation between reinfection and B.1.1.7 / total cases. 9

Table S3. Comparison of correlation between reinfection and B.1.1.7 / total cases. 10

COG-UK authorship list 11

Supplementary Figures

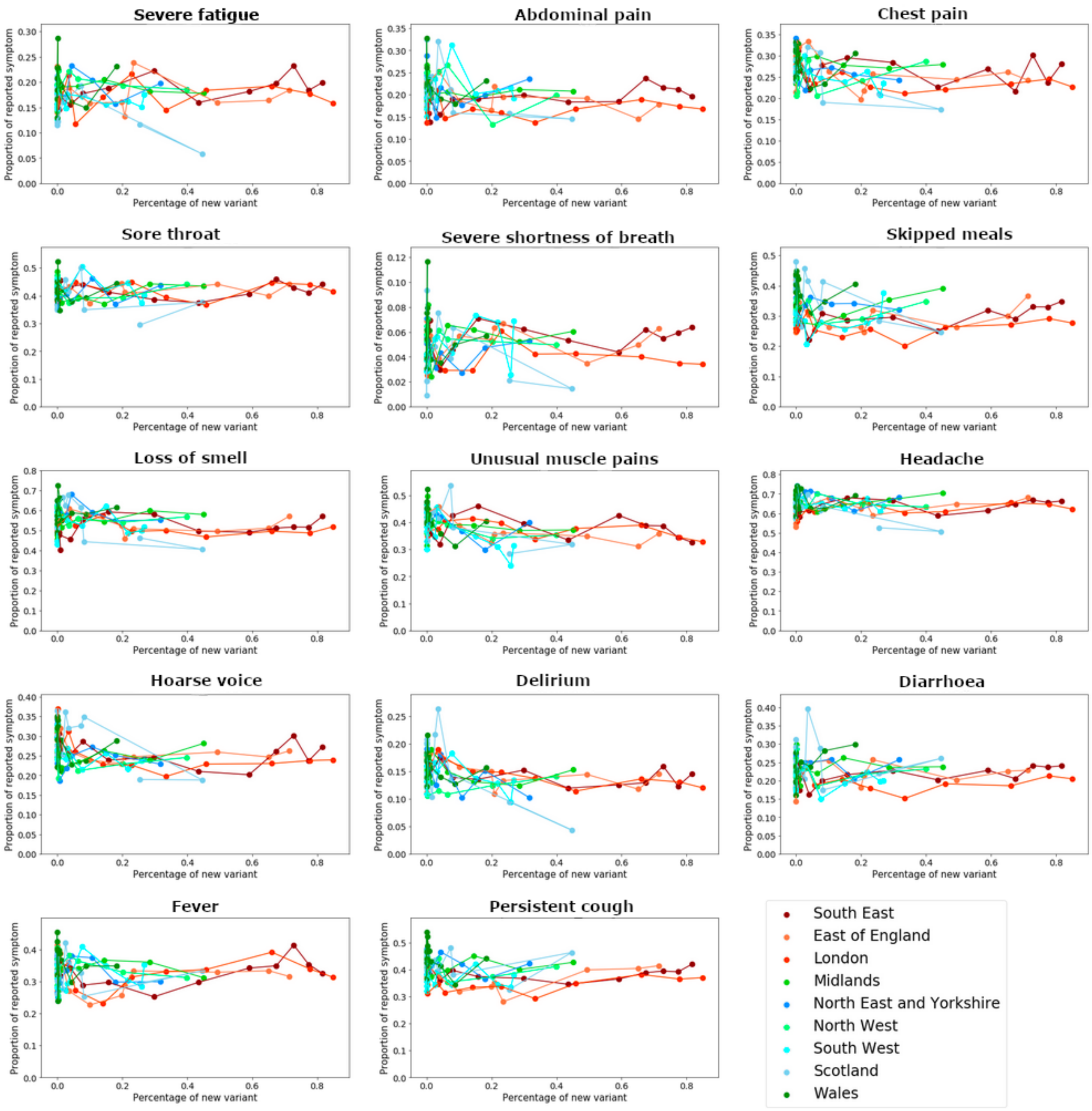

Figure S1. Symptom reporting against proportion of B.1.1.7.

Regional plots of the proportion of users with positive tests reporting each symptom, against the proportion of B.1.1.7. Drop in fever reporting in early November was caused by a change in the question wording; this wording was subsequently reverted a week later.

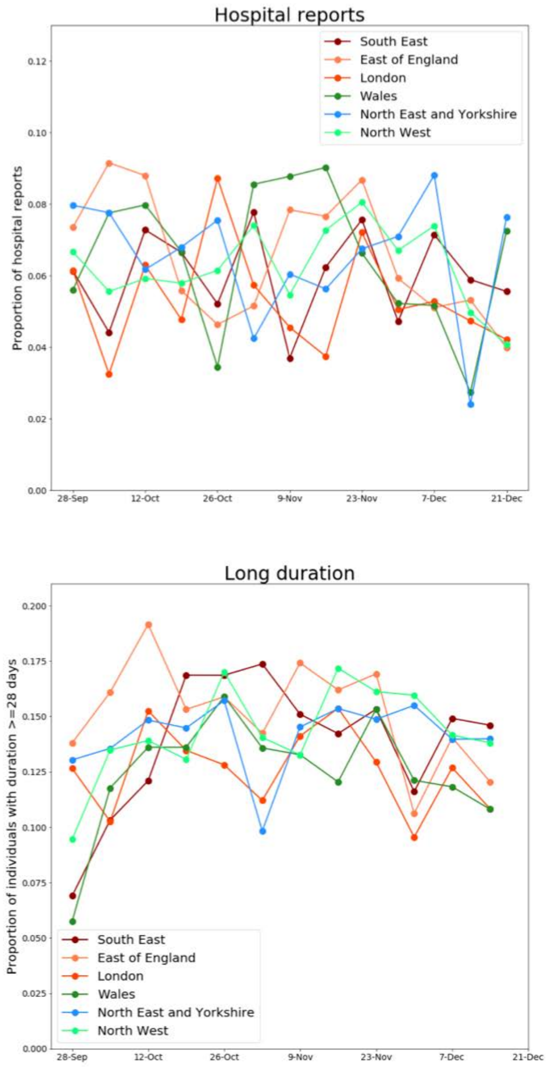

Figure S2. Hospitalisation, asymptomatics, long duration and total symptoms against time.

Regional plot of hospitalisation reports, proportion of asymptomatic positives, instances of long symptom duration and the total number of different experienced symptoms against time. For the study of long symptom duration, tests are only considered up to 21 December, and symptom reports up to 18 January 2021 to limit right censoring effects. Only symptomatic individuals for which duration can be ascertained are included.

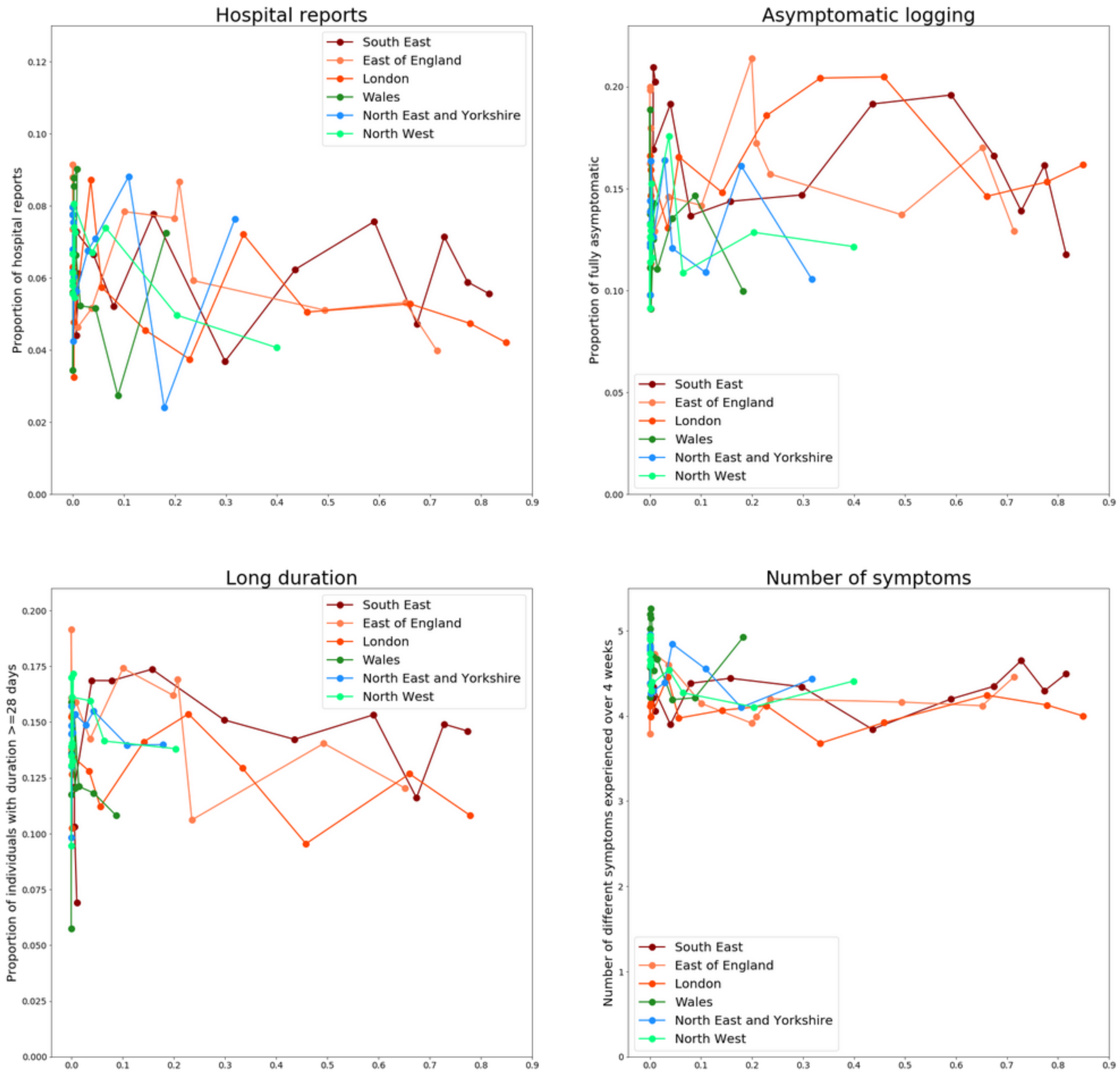

Figure S3. Hospitalisation, asymptomatics, long duration and total symptoms against proportion of B.1.1.7.

Regional plot of hospitalisation reports, proportion of asymptomatic positives, instances of long symptom duration and the total number of different experienced symptoms against proportion of B.1.1.7. For the study of long symptom duration, tests are only considered up to 21 December, and symptom reports up to 18 January 2021 to limit right censoring effects. Only symptomatic individuals for which duration can be ascertained are included.

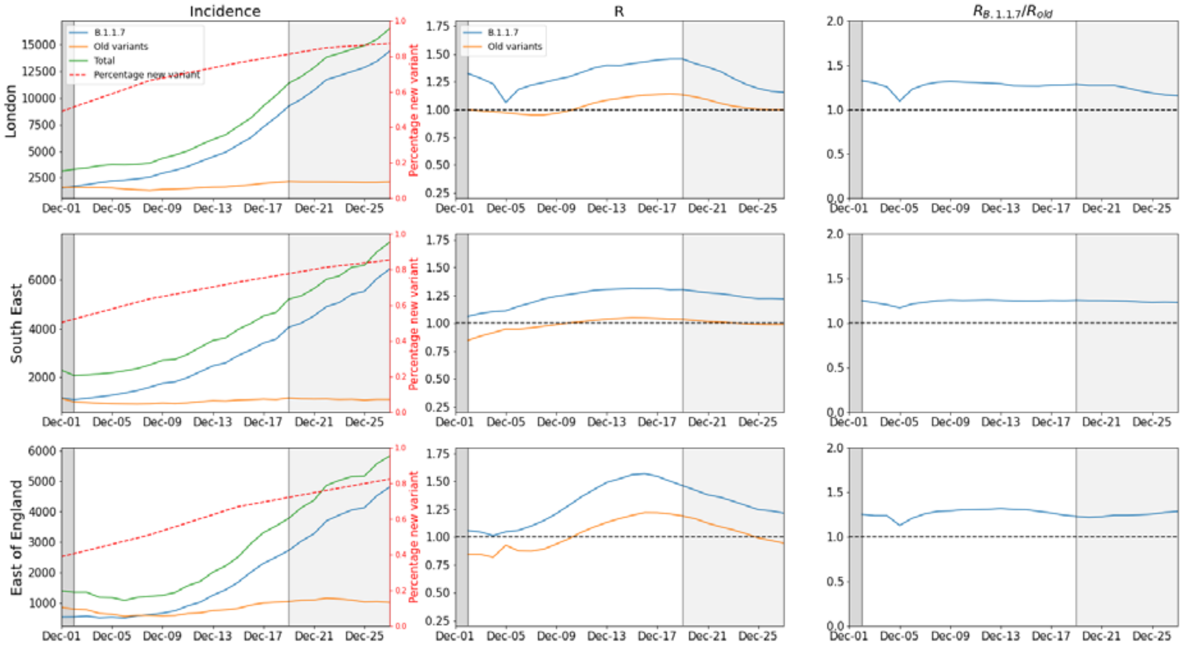

Figure S4. Incidence and R(t) using SGTF data.

Incidence and R(t) for the old and new variants, along with the ratio between these R values, for the three regions in England with the largest proportion of B.1.1.7, using SGTF data. Dark grey regions indicate national lockdowns, light grey shaded the period where London and much of the South East and East of England were placed in Tier 4 restrictions.

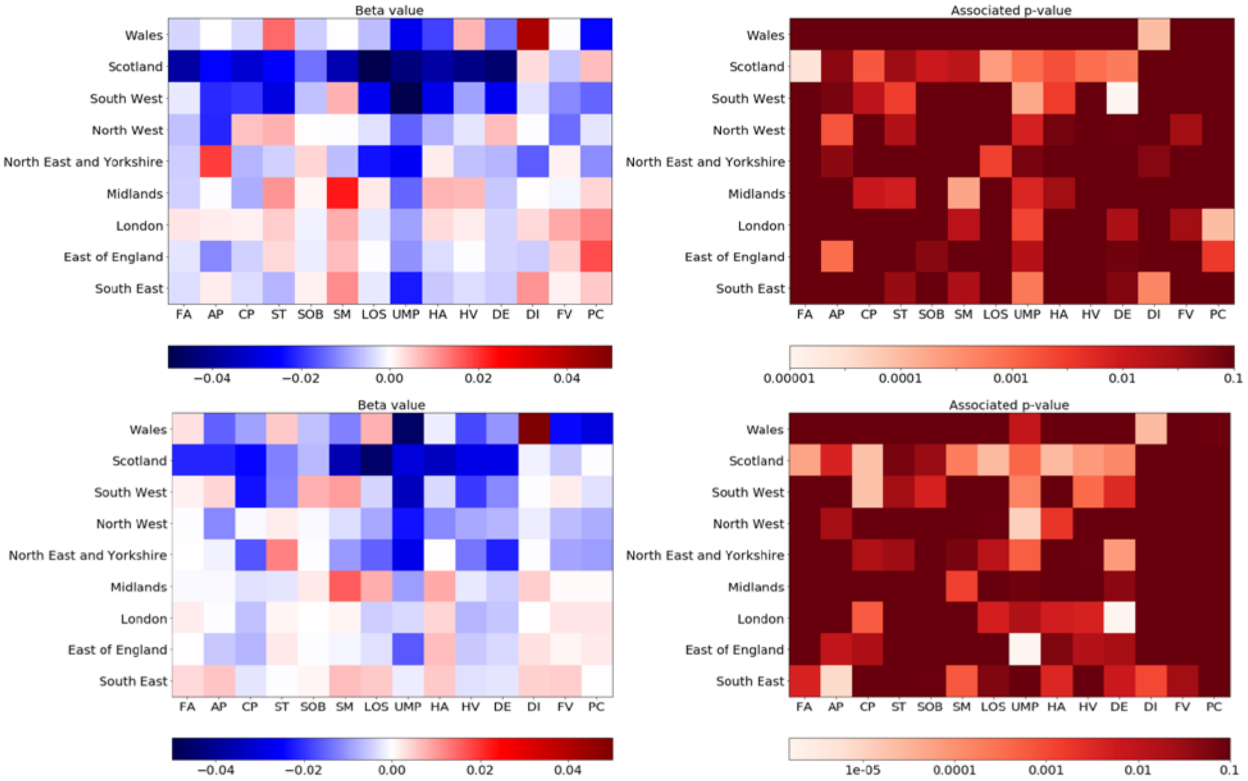

Figure S5. Associations between symptom reports and B.1.1.7.

Colour plot of beta values and associated p-values for each region and symptom when investigating association between symptom report (in a 4 week window around the test) and proportion of variant B.1.1.7. Top row shows results for uncorrected model, and the bottom row shows results for the model corrected for age, sex, temperature and humidity. Note that the p-values are capped at 0.1. Beta-values are presented for an increase of 0.1 in the proportion of variant B.1.1.7.

Key: FA - fatigue, AP - abdominal pain, CP - chest pain, ST - sore throat, SOB - shortness of breath, SM - skipped meals, LOS - loss of smell, UMP - unusual muscle pains, HA - headache, HV - hoarse voice, DE - delirium, DI - diarrhoea, FV - fever, PC - persistent cough

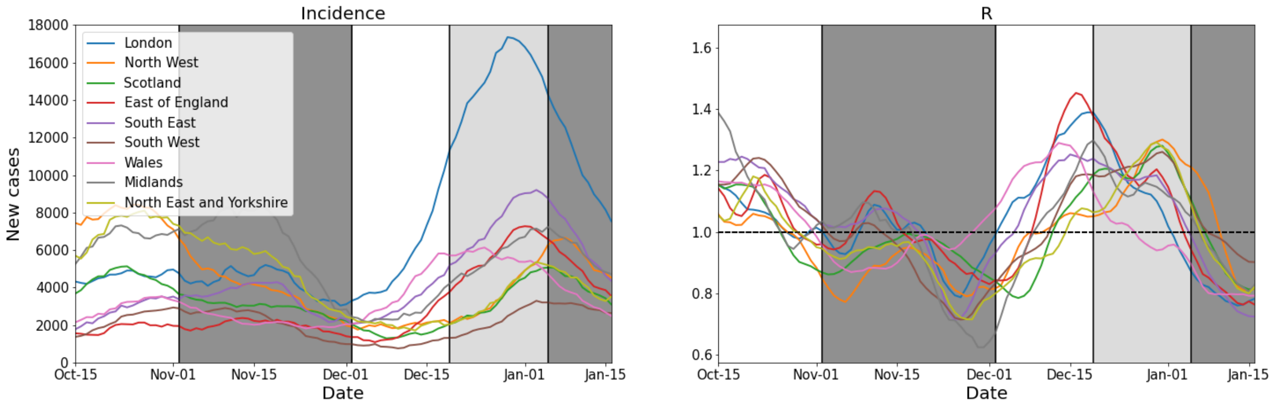

Figure S6. Incidence and R(t) for all regions, extended to 15 January 2021.

Total incidence and R(t) for all regions, extended to capture the third national lockdown beginning 5 January 2021. Dark grey regions indicate national lockdowns, light grey indicate the period where London and much of the South East and East of England were placed in Tier 4 restrictions.

Supplementary Tables

Table S1. Coefficients for association between B.1.1.7 and asymptomatic infection, long duration, hospital reports, number of symptoms.

Beta coefficient of the variant proportion when evaluating association with number of reported symptoms, asymptomatic rate, proportion of hospital report and proportion of individuals with symptom duration >=28 days across the different regions when correcting for age, sex, temperature and humidity. All values are presented for an increase in 0.1 in the proportion of variant B.1.1.7. All results are presented in the form mean [CI]; p-value

	Proportion of fully asymptomatic	Number of symptoms reported over 4 weeks around test	Proportion of hospital reports	Proportion of individuals with symptom duration >= 28days
South East	0.001 [-0.015;0.017] ; 0.901	-0.021 [-0.163;0.121] ; 0.733	-0.002 [-0.011;0.007] ; 0.624	-0.003 [-0.009;0.004] ; 0.37
East of England	0.002 [-0.008;0.012] ; 0.588	-0.012 [-0.153;0.13] ; 0.851	-0.002 [-0.01;0.006] ; 0.52	-0.002 [-0.015;0.01] ; 0.689
London	-0.005 [-0.014;0.005] ; 0.298	0.031 [-0.055;0.116] ; 0.423	-0.002 [-0.007;0.003] ; 0.298	-0.002 [-0.013;0.009] ; 0.682
Midlands	-0.016 [-0.028;-0.004] ; 0.014	0.02 [-0.133;0.173] ; 0.766	-0.002 [-0.007;0.003] ; 0.328	0.002 [-0.01;0.015] ; 0.671
North East and Yorkshire	-0.011 [-0.046;0.023] ; 0.462	-0.086 [-0.444;0.272] ; 0.586	-0.011 [-0.04;0.019] ; 0.426	0.015 [-0.022;0.052] ; 0.349
North West	-0.005 [-0.023;0.013] ; 0.512	-0.053 [-0.218;0.111] ; 0.468	-0.009 [-0.015;-0.004] ; 0.005	0 [-0.031;0.031] ; 0.98
South West	0.015 [-0.011;0.04] ; 0.217	-0.261 [-0.437;-0.085] ; 0.01	-0.001 [-0.02;0.018] ; 0.902	-0.048 [-0.091;-0.004] ; 0.036
Scotland	0.022 [-0.013;0.058] ;	-0.4 [-0.711;-0.088] ; 0.019	-0.018 [-0.037;0.002] ;	-0.012 [-0.027;0.003] ;

	0.177		0.073	0.107
Wales	-0.002 [-0.05;0.047] ; 0.943	-0.041 [-0.683;0.601] ; 0.884	-0.008 [-0.045;0.028] ; 0.602	-0.053 [-0.141;0.035] ; 0.192

Table S2. Regional reinfection rates and correlation between reinfection and B.1.1.7 / total cases.

Regional reinfection rates, and the Spearman correlation between daily reported reinfections and the proportion of B.1.1.7, and between the number of reinfections and new reported cases.

Region	Reinfection rate	Correlation between B.1.1.7 and reinfection	Correlation between new cases and reinfection
South East	1.3%	0.52	0.69
East of England	1.3%	0.56	0.60
London	0.9%	0.38	0.56
Midlands	1.3%	0.32	0.60
North East and Yorkshire	0.6%	-0.07	0.26
North West	0.8%	-0.01	0.34
South West	0.9%	-0.16	0.27
Scotland	0.6%	0.38	-0.13
Wales	1.0%	0.12	0.32

Table S3. Comparison of correlation between reinfection and B.1.1.7 / total cases.

Comparison of regional correlation over time between proportion of B.1.1.7 and number of possible reinfections and between new reported cases and number of possible reinfections. Medians over 100 bootstrapped samples are calculated for each and compared using a Mann-Whitney U test.

Region	Median correlation between B.1.1.7 and reinfection	Median correlation between new cases and reinfection	p-value
South East	0.55	0.69	<0.001
East of England	0.51	0.56	<0.001
London	0.46	0.62	<0.001
Midlands	0.28	0.75	<0.001
North East and Yorkshire	-0.02	0.30	<0.001
North West	0.06	0.43	<0.001
South West	-0.35	0.05	<0.001
Scotland	0.59	-0.15	<0.001
Wales	0.07	0.26	<0.001

COG-UK authorship list

Funding acquisition, Leadership and supervision, Metadata curation, Project administration, Samples and logistics, Sequencing and analysis, Software and analysis tools, and Visualisation:
Samuel C Robson [13].

Funding acquisition, Leadership and supervision, Metadata curation, Project administration, Samples and logistics, Sequencing and analysis, and Software and analysis tools:
Nicholas J Loman [41] and Thomas R Connor [10, 69].

Leadership and supervision, Metadata curation, Project administration, Samples and logistics, Sequencing and analysis, Software and analysis tools, and Visualisation:
Tanya Golubchik [5].

Funding acquisition, Metadata curation, Samples and logistics, Sequencing and analysis, Software and analysis tools, and Visualisation:
Rocio T Martinez Nunez [42].

Funding acquisition, Leadership and supervision, Metadata curation, Project administration, and Samples and logistics:
Catherine Ludden [88].

Funding acquisition, Leadership and supervision, Metadata curation, Samples and logistics, and Sequencing and analysis:
Sally Corden [69].

Funding acquisition, Leadership and supervision, Project administration, Samples and logistics, and Sequencing and analysis:
Ian Johnston [99] and David Bonsall [5].

Funding acquisition, Leadership and supervision, Sequencing and analysis, Software and analysis tools, and Visualisation:
Colin P Smith [87] and Ali R Awan [28].

Funding acquisition, Samples and logistics, Sequencing and analysis, Software and analysis tools, and Visualisation:
Giselda Bucca [87].

Leadership and supervision, Metadata curation, Project administration, Samples and logistics, and Sequencing and analysis:
M. Estee Torok [22, 101].

Leadership and supervision, Metadata curation, Project administration, Samples and logistics, and Visualisation:
Kordo Saeed [81, 110] and Jacqui A Prieto [83, 109].

Leadership and supervision, Metadata curation, Project administration, Sequencing and analysis, and Software and analysis tools:

David K Jackson [99].

Metadata curation, Project administration, Samples and logistics, Sequencing and analysis, and Software and analysis tools:
William L Hamilton [22].
Metadata curation, Project administration, Samples and logistics, Sequencing and analysis, and Visualisation:
Luke B Snell [11].

Funding acquisition, Leadership and supervision, Metadata curation, and Samples and logistics:
Catherine Moore [69].

Funding acquisition, Leadership and supervision, Project administration, and Samples and logistics:
Ewan M Harrison [99, 88].

Leadership and supervision, Metadata curation, Project administration, and Samples and logistics:
Sonia Goncalves [99].

Leadership and supervision, Metadata curation, Samples and logistics, and Sequencing and analysis:
Ian G Goodfellow [24], Derek J Fairley [3, 72], Matthew W Loose [18] and Joanne Watkins [69].

Leadership and supervision, Metadata curation, Samples and logistics, and Software and analysis tools:
Rich Livett [99].

Leadership and supervision, Metadata curation, Samples and logistics, and Visualisation:
Samuel Moses [25, 106].

Leadership and supervision, Metadata curation, Sequencing and analysis, and Software and analysis tools:
Roberto Amato [99], Sam Nicholls [41] and Matthew Bull [69].

Leadership and supervision, Project administration, Samples and logistics, and Sequencing and analysis:
Darren L Smith [37, 58, 105].

Leadership and supervision, Sequencing and analysis, Software and analysis tools, and Visualisation:
Jeff Barrett [99], David M Aanensen [14, 114].

Metadata curation, Project administration, Samples and logistics, and Sequencing and analysis:
Martin D Curran [65], Surendra Parmar [65], Dinesh Aggarwal [95, 99, 64] and James G Shepherd [48].

Metadata curation, Project administration, Sequencing and analysis, and Software and analysis tools:
Matthew D Parker [93].

Metadata curation, Samples and logistics, Sequencing and analysis, and Visualisation:
Sharon Glaysher [61].

Metadata curation, Sequencing and analysis, Software and analysis tools, and Visualisation:
Matthew Bashton [37, 58], Anthony P Underwood [14, 114], Nicole Pacchiarini [69] and Katie F Loveson [77].

Project administration, Sequencing and analysis, Software and analysis tools, and Visualisation:
Alessandro M Carabelli [88].

Funding acquisition, Leadership and supervision, and Metadata curation:
Kate E Templeton [53, 90].

Funding acquisition, Leadership and supervision, and Project administration:
Cordelia F Langford [99], John Sillitoe [99], Thushan I de Silva [93] and Dennis Wang [93].

Funding acquisition, Leadership and supervision, and Sequencing and analysis:
Dominic Kwiatkowski [99, 107], **And**rew Rambaut [90], Justin O'Grady [70, 89] and Simon Cottrell [69].

Leadership and supervision, Metadata curation, and Sequencing and analysis:
Matthew T.G. Holden [68] and Emma C Thomson [48].

Leadership and supervision, Project administration, and Samples and logistics:
Husam Osman [64, 36], Monique Andersson [59], Anoop J Chauhan [61] and Mohammed O Hassan-Ibrahim [6].

Leadership and supervision, Project administration, and Sequencing and analysis:
Mara Lawniczak [99].

Leadership and supervision, Samples and logistics, and Sequencing and analysis:
Ravi Kumar Gupta [88, 113], Alex Alderton [99], Meera Chand [66], Chrystala Constantinidou [94], Meera Unnikrishnan [94], Alistair C Darby [92], Julian A Hiscox [92] and Steve Paterson [92].

Leadership and supervision, Sequencing and analysis, and Software and analysis tools:
Inigo Martincorena [99], David L Robertson [48], Erik M Volz [39], Andrew J Page [70] and Oliver G Pybus [23].

Leadership and supervision, Sequencing and analysis, and Visualisation:
Andrew R Bassett [99].

Metadata curation, Project administration, and Samples and logistics:
Cristina V Ariani [99], Michael H Spencer Chapman [99, 88], Kathy K Li [48], Rajiv N Shah [48], Natasha G Jesudason [48] and Yusri Taha [50].

Metadata curation, Project administration, and Sequencing and analysis:
Martin P McHugh [53] and Rebecca Dewar [53].

Metadata curation, Samples and logistics, and Sequencing and analysis:
Aminu S Jahun [24], Claire McMurray [41], Sarojini Pandey [84], James P McKenna [3], Andrew Nelson [58, 105], Gregory R Young [37, 58], Clare M McCann [58, 105] and Scott Elliott [61].

Metadata curation, Samples and logistics, and Visualisation:
Hannah Lowe [25].

Metadata curation, Sequencing and analysis, and Software and analysis tools:
Ben Temperton [91], Sunando Roy [82], Anna Price [10], Sara Rey [69] and Matthew Wyles [93].

Metadata curation, Sequencing and analysis, and Visualisation:
Stefan Rooke [90] and Sharif Shaaban [68].

Project administration, Samples and logistics, Sequencing and analysis:
Mariateresa de Cesare [98].

Project administration, Samples and logistics, and Software and analysis tools:
Laura Letchford [99].

Project administration, Samples and logistics, and Visualisation:
Siona Silveira [81], Emanuela Pelosi [81] and Eleri Wilson-Davies [81].

Samples and logistics, Sequencing and analysis, and Software and analysis tools:
Myra Hosmillo [24].

Sequencing and analysis, Software and analysis tools, and Visualisation:
Áine O'Toole [90], Andrew R Hesketh [87], Richard Stark [94], Louis du Plessis [23], Chris Ruis [88], Helen Adams [4] and Yann Bourgeois [76].

Funding acquisition, and Leadership and supervision:
Stephen L Michell [91], Dimitris Grammatopoulos[84, 112], Jonathan Edgeworth [12], Judith Breuer [30, 82], John A Todd [98] and Christophe Fraser [5].

Funding acquisition, and Project administration:
David Buck [98] and Michaela John [9].

Leadership and supervision, and Metadata curation:
Gemma L Kay [70].

Leadership and supervision, and Project administration:
Steve Palmer [99], Sharon J Peacock [88, 64] and David Heyburn [69].

Leadership and supervision, and Samples and logistics:
Danni Weldon [99], Esther Robinson [64, 36], Alan McNally [41, 86], Peter Muir [64], Ian B Vipond [64], John BoYes [29], Venkat Sivaprakasam [46], Tranprit Salluja [75], Samir Dervisevic [54] and Emma J Meader [54].

Leadership and supervision, and Sequencing and analysis:
Naomi R Park [99], Karen Oliver [99], Aaron R Jeffries [91], Sascha Ott [94], Ana da Silva Filipe [48], David A Simpson [72] and Chris Williams [69].

Leadership and supervision, and Visualisation:

Jane AH Masoli [73, 91].

Metadata curation, and Samples and logistics:
Bridget A Knight [73, 91], Christopher R Jones [73, 91], Cherian Koshy [1], Amy Ash [1], Anna Casey [71], Andrew Bosworth [64, 36], Liz Ratcliffe [71], Li Xu-McCrae [36], Hannah M Pymont [64], Stephanie Hutchings [64], Lisa Berry [84], Katie Jones [84], Fenella Halstead [46], Thomas Davis [21], Christopher Holmes [16], Miren Iturriza-Gomara [92], Anita O Lucaci [92], Paul Anthony Randell [38, 104], Alison Cox [38, 104], Pinglawathee Madona [38, 104], Kathryn Ann Harris [30], Julianne Rose Brown [30], Tabitha W Mahungu [74], Dianne Irish-Tavares [74], Tanzina Haque [74], Jennifer Hart [74], Eric Witele [74], Melisa Louise Fenton [75], Steven Liggett [79], Clive Graham [56], Emma Swindells [57], Jennifer Collins [50], Gary Eltringham [50], Sharon Campbell [17], Patrick C McClure [97], Gemma Clark [15], Tim J Sloan [60], Carl Jones [15] and Jessica Lynch [2, 111].

Metadata curation, and Sequencing and analysis:
Ben Warne [8], Steven Leonard [99], Jillian Durham [99], Thomas Williams [90], Sam T Haldenby [92], Nathaniel Storey [30], Nabil-Fareed Alikhan [70], Nadine Holmes [18], Christopher Moore [18], Matthew Carlile [18], Malorie Perry [69], Noel Craine [69], Ronan A Lyons [80], Angela H Beckett [13], Salman Goudarzi [77], Christopher Fearn [77], Kate Cook [77], Hannah Dent [77] and Hannah Paul [77].

Metadata curation, and Software and analysis tools:
Robert Davies [99].

Project administration, and Samples and logistics:
Beth Blane [88], Sophia T Girgis [88], Mathew A Beale [99], Katherine L Bellis [99, 88], Matthew J Dorman [99], Eleanor Drury [99], Leanne Kane [99], Sally Kay [99], Samantha McGuigan [99], Rachel Nelson [99], Liam Prestwood [99], Shavanthi Rajatileka [99], Rahul Batra [12], Rachel J Williams [82], Mark Kristiansen [82], Angie Green [98], Anita Justice [59], Adhyana I.K Mahanama [81, 102] and Buddhini Samaraweera [81, 102].

Project administration, and Sequencing and analysis:
Nazreen F Hadjirin [88] and Joshua Quick [41].

Project administration, and Software and analysis tools:
Radoslaw Poplawski [41].

Samples and logistics, and Sequencing and analysis:
Leanne M Kermack [88], Nicola Reynolds [7], Grant Hall [24], Yasmin Chaudhry [24], Malte L Pinckert [24], Iliana Georgana [24], Robin J Moll [99], Alicia Thornton [66], Richard Myers [66], Joanne Stockton [41], Charlotte A Williams [82], Wen C Yew [58], Alexander J Trotter [70], Amy Trebes [98], George MacIntyre-Cockett [98], Alec Birchley [69], Alexander Adams [69], Amy Plimmer [69], Bree Gatica-Wilcox [69], Caoimhe McKerr [69], Ember Hilvers [69], Hannah Jones [69], Hibo Asad [69], Jason Coombes [69], Johnathan M Evans [69], Laia Fina [69], Lauren Gilbert [69], Lee Graham [69], Michelle Cronin [69], Sara Kumziene-SummerhaYes [69], Sarah Taylor [69], Sophie Jones [69], Danielle C Groves [93], Peijun Zhang [93], Marta Gallis [93] and Stavroula F Louka [93].

Samples and logistics, and Software and analysis tools:
Igor Starinskij [48].

Sequencing and analysis, and Software and analysis tools:

Chris J Illingworth [47], Chris Jackson [47], Marina Gourtovaia [99], Gerry Tonkin-Hill [99], Kevin Lewis [99], Jaime M Tovar-Corona [99], Keith James [99], Laura Baxter [94], Mohammad T. Alam [94], Richard J Orton [48], Joseph Hughes [48], Sreenu Vattipally [48], Manon Ragonnet-Cronin [39], Fabricia F. Nascimento [39], David Jorgensen [39], Olivia Boyd [39], Lily Geidelberg [39], Alex E Zarebski [23], Jayna Raghwani [23], Moritz UG Kraemer [23], Joel Southgate [10, 69], Benjamin B Lindsey [93] and Timothy M Freeman [93].

Software and analysis tools, and Visualisation:
Jon-Paul Keatley [99], Joshua B Singer [48], Leonardo de Oliveira Martins [70], Corin A Yeats [14], Khalil Abudahab [14, 114], Ben EW Taylor [14, 114] and Mirko Menegazzo [14].

Leadership and supervision:
John Danesh [99], Wendy Hogsden [46], Sahar Eldirdiri [21], Anita Kenyon [21], Jenifer Mason [43], Trevor I Robinson [43], Alison Holmes [38, 103], James Price [38, 103], John A Hartley [82], Tanya Curran [3], Alison E Mather [70], Giri Shankar [69], Rachel Jones [69], Robin Howe [69] and Sian Morgan [9].

Metadata curation:
Elizabeth Wastenge [53], Michael R Chapman [34, 88, 99], Siddharth Mookerjee [38, 103], Rachael Stanley [54], Wendy Smith [15], Timothy Peto [59], David Eyre [59], Derrick Crook [59], Gabrielle Vernet [33], Christine Kitchen [10], Huw Gulliver [10], Ian Merrick [10], Martyn Guest [10], Robert Munn [10], Declan T Bradley [63, 72] and Tim Wyatt [63].

Project administration:
Charlotte Beaver [99], Luke Foulser [99], Sophie Palmer [88], Carol M Churcher [88], Ellena Brooks [88], Kim S Smith [88], Katerina Galai [88], Georgina M McManus [88], Frances Bolt [38, 103], Francesc Coll [19], Lizzie Meadows [70], Stephen W Attwood [23], Alisha Davies [69], Elen De Lacy [69], Fatima Downing [69], Sue Edwards [69], Garry P Scarlett [76], Sarah Jeremiah [83] and Nikki Smith [93].

Samples and logistics:
Danielle Leek [88], Sushmita Sridhar [88, 99], Sally Forrest [88], Claire Cormie [88], Harmeet K Gill [88], Joana Dias [88], Ellen E Higginson [88], Mailis Maes [88], Jamie Young [88], Michelle Wantoch [7], Sanger Covid Team (www.sanger.ac.uk/covid-team) [99], Dorota Jamrozy [99], Stephanie Lo [99], Minal Patel [99], Verity Hill [90], Claire M Bewshea [91], Sian Ellard [73, 91], Cressida Auckland [73], Ian Harrison [66], Chloe Bishop [66], Vicki Chalker [66], Alex Richter [85], Andrew Beggs [85], Angus Best [86], Benita Percival [86], Jeremy Mirza [86], Oliver Megram [86], Megan Mayhew [86], Liam Crawford [86], Fiona Ashcroft [86], Emma Moles-Garcia [86], Nicola Cumley [86], Richard Hopes [64], Patawee Asamaphan [48], Marc O Niebel [48], Rory N Gunson [100], Amanda Bradley [52], Alasdair Maclean [52], Guy Mollett [52], Rachel Blacow [52], Paul Bird [16], Thomas Helmer [16], Karlie Fallon [16], Julian Tang [16], Antony D Hale [49], Louissa R Macfarlane-Smith [49], Katherine L Harper [49], Holli Carden [49], Nicholas W Machin [45, 64], Kathryn A Jackson [92], Shazaad S Y Ahmad [45, 64], Ryan P George [45], Lance Turtle [92], Elaine O'Toole [43], Joanne Watts [43], Cassie Breen [43], Angela Cowell [43], Adela Alcolea-Medina [32, 96], Themoula Charalampous [12, 42], Amita Patel [11], Lisa J Levett [35], Judith Heaney [35], Aileen Rowan [39], Graham P Taylor [39], Divya Shah [30], Laura Atkinson [30], Jack CD Lee [30], Adam P Westhorpe [82], Riaz Jannoo [82], Helen L Lowe [82], Angeliki Karamani [82], Leah Ensell [82], Wendy Chatterton [35], Monika Pusok [35], Ashok Dadrah [75], Amanda Symmonds [75], Graciela Sluga [44], Zoltan Molnar [72], Paul Baker [79], Stephen Bonner [79], Sarah Essex [79], Edward Barton [56], Debra Padgett [56], Garren Scott [56], Jane Greenaway [57], Brendan AI Payne [50], Shirelle Burton-Fanning [50], Sheila Waugh [50], Veena Raviprakash [17], Nicola Sheriff [17], Victoria Blakey [17], Lesley-Anne Williams [17], Jonathan Moore [27], Susanne Stonehouse [27], Louise Smith [55], Rose K Davidson [89], Luke Bedford [26], Lindsay Coupland [54], Victoria Wright [18], Joseph G Chappell [97], Theocharis Tsoleridis [97], Jonathan Ball [97], Manjinder Khakh [15], Vicki M Fleming [15], Michelle M Lister [15], Hannah C Howson-Wells [15], Louise Berry [15], Tim Boswell [15], Amelia Joseph [15], Iona Willingham [15], Nichola Duckworth [60], Sarah

Walsh [60], Emma Wise [2, 111], Nathan Moore [2, 111], Matilde Mori [2, 108, 111], Nick Cortes [2, 111], Stephen Kidd [2, 111], Rebecca Williams [33], Laura Gifford [69], Kelly Bicknell [61], Sarah Wyllie [61], Allyson Lloyd [61], Robert Impey [61], Cassandra S Malone [6], Benjamin J Cogger [6], Nick Levene [62], Lynn Monaghan [62], Alexander J Keeley [93], David G Partridge [78, 93], Mohammad Raza [78, 93], Cariad Evans [78, 93] and Kate Johnson [78, 93].

Sequencing and analysis:
Emma Betteridge [99], Ben W Farr [99], Scott Goodwin [99], Michael A Quail [99], Carol Scott [99], Lesley Shirley [99], Scott AJ Thurston [99], Diana Rajan [99], Iraad F Bronner [99], Louise Aigrain [99], Nicholas M Redshaw [99], Stefanie V Lensing [99], Shane McCarthy [99], Alex Makunin [99], Carlos E Balcazar [90], Michael D Gallagher [90], Kathleen A Williamson [90], Thomas D Stanton [90], Michelle L Michelsen [91], Joanna Warwick-Dugdale [91], Robin Manley [91], Audrey Farbos [91], James W Harrison [91], Christine M Sambles [91], David J Studholme [91], Angie Lackenby [66], Tamyo Mbisa [66], Steven Platt [66], Shahjahan Miah [66], David Bibby [66], Carmen Manso [66], Jonathan Hubb [66], Gavin Dabrera [66], Mary Ramsay [66], Daniel Bradshaw [66], Ulf Schaefer [66], Natalie Groves [66], Eileen Gallagher [66], David Lee [66], David Williams [66], Nicholas Ellaby [66], Hassan Hartman [66], Nikos Manesis [66], Vineet Patel [66], Juan Ledesma [67], Katherine A Twohig [67], Elias Allara [64, 88], Clare Pearson [64, 88], Jeffrey K. J. Cheng [94], Hannah E. Bridgewater [94], Lucy R. Frost [94], Grace Taylor-Joyce [94], Paul E Brown [94], Lily Tong [48], Alice Broos [48], Daniel Mair [48], Jenna Nichols [48], Stephen N Carmichael [48], Katherine L Smollett [40], Kyriaki Nomikou [48], Elihu Aranday-Cortes [48], Natasha Johnson [48], Seema Nickbakhsh [48, 68], Edith E Vamos [92], Margaret Hughes [92], Lucille Rainbow [92], Richard Eccles [92], Charlotte Nelson [92], Mark Whitehead [92], Richard Gregory [92], Matthew Gemmell [92], Claudia Wierzbicki [92], Hermione J Webster [92], Chloe L Fisher [28], Adrian W Signell [20], Gilberto Betancor [20], Harry D Wilson [20], Gaia Nebbia [12], Flavia Flaviani [31], Alberto C Cerda [96], Tammy V Merrill [96], Rebekah E Wilson [96], Marius Cotic [82], Nadua Bayzid [82], Thomas Thompson [72], Erwan Acheson [72], Steven Rushton [51], Sarah O'Brien [51], David J Baker [70], Steven Rudder [70], Alp Aydin [70], Fei Sang [18], Johnny Debebe [18], Sarah Francois [23], Tetyana I Vasylyeva [23], Marina Escalera Zamudio [23], Bernardo Gutierrez [23], Angela Marchbank [10], Joshua Maksimovic [9], Karla Spellman [9], Kathryn McCluggage [9], Mari Morgan [69], Robert Beer [9], Safiah Afifi [9], Trudy Workman [10], William Fuller [10], Catherine Bresner [10], Adrienn Angyal [93], Luke R Green [93], Paul J Parsons [93], Rachel M Tucker [93], Rebecca Brown [93] and Max Whiteley [93].

Software and analysis tools:
James Bonfield [99], Christoph Puethe [99], Andrew Whitwham [99], Jennifier Liddle [99], Will Rowe [41], Igor Siveroni [39], Thanh Le-Viet [70] and Amy Gaskin [69].

Visualisation:
Rob Johnson [39].

1 Barking, Havering and Redbridge University Hospitals NHS Trust, **2** Basingstoke Hospital, **3** Belfast Health & Social Care Trust, **4** Betsi Cadwaladr University Health Board, **5** Big Data Institute, Nuffield Department of Medicine, University of Oxford, **6** Brighton and Sussex University Hospitals NHS Trust, **7** Cambridge Stem Cell Institute, University of Cambridge, **8** Cambridge University Hospitals NHS Foundation Trust, **9** Cardiff and Vale University Health Board, **10** Cardiff University, **11** Centre for Clinical Infection & Diagnostics Research, St. Thomas' Hospital and Kings College London, **12** Centre for Clinical Infection and Diagnostics Research, Department of Infectious Diseases, Guy's and St Thomas' NHS Foundation Trust, **13** Centre for Enzyme Innovation, University of Portsmouth (PORT), **14** Centre for Genomic Pathogen Surveillance, University of Oxford, **15** Clinical Microbiology Department, Queens Medical Centre, **16** Clinical Microbiology, University Hospitals of Leicester NHS Trust, **17** County Durham and Darlington NHS Foundation Trust, **18** Deep Seq, School of Life Sciences, Queens Medical Centre,

University of Nottingham, **19** Department of Infection Biology, Faculty of Infectious & Tropical Diseases, London School of Hygiene & Tropical Medicine, **20** Department of Infectious Diseases, King's College London, **21** Department of Microbiology, Kettering General Hospital, **22** Departments of Infectious Diseases and Microbiology, Cambridge University Hospitals NHS Foundation Trust; Cambridge, UK, **23** Department of Zoology, University of Oxford, **24** Division of Virology, Department of Pathology, University of Cambridge, **25** East Kent Hospitals University NHS Foundation Trust, **26** East Suffolk and North Essex NHS Foundation Trust, **27** Gateshead Health NHS Foundation Trust, **28** Genomics Innovation Unit, Guy's and St. Thomas' NHS Foundation Trust, **29** Gloucestershire Hospitals NHS Foundation Trust, **30** Great Ormond Street Hospital for Children NHS Foundation Trust, **31** Guy's and St. Thomas' BRC, **32** Guy's and St. Thomas' Hospitals, **33** Hampshire Hospitals NHS Foundation Trust, **34** Health Data Research UK Cambridge, **35** Health Services Laboratories, **36** Heartlands Hospital, Birmingham, **37** Hub for Biotechnology in the Built Environment, Northumbria University, **38** Imperial College Hospitals NHS Trust, **39** Imperial College London, **40** Institute of Biodiversity, Animal Health & Comparative Medicine, **41** Institute of Microbiology and Infection, University of Birmingham, **42** King's College London, **43** Liverpool Clinical Laboratories, **44** Maidstone and Tunbridge Wells NHS Trust, **45** Manchester University NHS Foundation Trust, **46** Microbiology Department, Wye Valley NHS Trust, Hereford, **47** MRC Biostatistics Unit, University of Cambridge, **48** MRC-University of Glasgow Centre for Virus Research, **49** National Infection Service, PHE and Leeds Teaching Hospitals Trust, **50** Newcastle Hospitals NHS Foundation Trust, **51** Newcastle University, **52** NHS Greater Glasgow and Clyde, **53** NHS Lothian, **54** Norfolk and Norwich University Hospital, **55** Norfolk County Council, **56** North Cumbria Integrated Care NHS Foundation Trust, **57** North Tees and Hartlepool NHS Foundation Trust, **58** Northumbria University, **59** Oxford University Hospitals NHS Foundation Trust, **60** PathLinks, Northern Lincolnshire & Goole NHS Foundation Trust, **61** Portsmouth Hospitals University NHS Trust, **62** Princess Alexandra Hospital Microbiology Dept., **63** Public Health Agency, **64** Public Health England, **65** Public Health England, Clinical Microbiology and Public Health Laboratory, Cambridge, UK, **66** Public Health England, Colindale, **67** Public Health England, Colindale, **68** Public Health Scotland, **69** Public Health Wales NHS Trust, **70** Quadram Institute Bioscience, **71** Queen Elizabeth Hospital, **72** Queen's University Belfast, **73** Royal Devon and Exeter NHS Foundation Trust, **74** Royal Free NHS Trust, **75** Sandwell and West Birmingham NHS Trust, **76** School of Biological Sciences, University of Portsmouth (PORT), **77** School of Pharmacy and Biomedical Sciences, University of Portsmouth (PORT), **78** Sheffield Teaching Hospitals, **79** South Tees Hospitals NHS Foundation Trust, **80** Swansea University, **81** University Hospitals Southampton NHS Foundation Trust, **82** University College London, **83** University Hospital Southampton NHS Foundation Trust, **84** University Hospitals Coventry and Warwickshire, **85** University of Birmingham, **86** University of Birmingham Turnkey Laboratory, **87** University of Brighton, **88** University of Cambridge, **89** University of East Anglia, **90** University of Edinburgh, **91** University of Exeter, **92** University of Liverpool, **93** University of Sheffield, **94** University of Warwick, **95** University of Cambridge, **96** Viapath, Guy's and St Thomas' NHS Foundation Trust, and King's College Hospital NHS Foundation Trust, **97** Virology, School of Life Sciences, Queens Medical Centre, University of Nottingham, **98** Wellcome Centre for Human Genetics, Nuffield Department of Medicine, University of Oxford, **99** Wellcome Sanger Institute, **100** West of Scotland Specialist Virology Centre, NHS Greater Glasgow and Clyde, **101** Department of Medicine, University of Cambridge, **102** Ministry of Health, Sri Lanka, **103** NIHR Health Protection Research Unit in HCAI and AMR, Imperial College London, **104** North West London Pathology, **105** NU-OMICS, Northumbria University, **106** University of Kent, **107** University of Oxford, **108** University of Southampton, **109** University of Southampton School of Health Sciences, **110** University of Southampton School of Medicine, **111** University of Surrey, **112** Warwick Medical School and Institute of Precision Diagnostics, Pathology, UHCW NHS Trust, **113** Wellcome Africa Health Research Institute Durban and **114** Wellcome Genome Campus.
